# Decellularized Allogeneic Heart Valves Demonstrate Self-Regeneration Potential after a Long-Term Preclinical Evaluation

**DOI:** 10.1371/journal.pone.0099593

**Published:** 2014-06-18

**Authors:** Laura Iop, Antonella Bonetti, Filippo Naso, Stefania Rizzo, Stefano Cagnin, Roberto Bianco, Carlo Dal Lin, Paolo Martini, Helen Poser, Paolo Franci, Gerolamo Lanfranchi, Roberto Busetto, Michel Spina, Cristina Basso, Maurizio Marchini, Alessandro Gandaglia, Fulvia Ortolani, Gino Gerosa

**Affiliations:** 1 Department of Cardiac, Thoracic and Vascular Sciences, University of Padua, Padua, Italy; 2 Department of Experimental and Clinical Medicine, University of Udine, Udine, Italy; 3 Department of Biology and C.R.I.B.I. Biotechnology Centre, University of Padua, Padua, Italy; 4 Department of Animal Medicine, Productions and Health, University of Padua, Legnaro, Italy; 5 Department of Biomedical Sciences, University of Padua, Padua, Italy; Foundation for Applied Medical research, Spain

## Abstract

Tissue-engineered heart valves are proposed as novel viable replacements granting longer durability and growth potential. However, they require extensive *in vitro* cell-conditioning in bioreactor before implantation. Here, the propensity of non-preconditioned decellularized heart valves to spontaneous in body self-regeneration was investigated in a large animal model. Decellularized porcine aortic valves were evaluated for right ventricular outflow tract (RVOT) reconstruction in Vietnamese Pigs (n = 11) with 6 (n = 5) and 15 (n = 6) follow-up months. Repositioned native valves (n = 2 for each time) were considered as control. Tissue and cell components from explanted valves were investigated by histology, immunohistochemistry, electron microscopy, and gene expression. Most substitutes constantly demonstrated *in vivo* adequate hemodynamic performances and *ex vivo* progressive repopulation during the 15 implantation months without signs of calcifications, fibrosis and/or thrombosis, as revealed by histological, immunohistochemical, ultrastructural, metabolic and transcriptomic profiles. Colonizing cells displayed native-like phenotypes and actively synthesized novel extracellular matrix elements, as collagen and elastin fibers. New mature blood vessels, i.e. capillaries and *vasa vasorum*, were identified in repopulated valves especially in the medial and adventitial *tunicae* of regenerated arterial walls. Such findings correlated to the up-regulated vascular gene transcription. Neoinnervation hallmarks were appreciated at histological and ultrastructural levels. Macrophage populations with reparative M2 phenotype were highly represented in repopulated valves. Indeed, no aspects of adverse/immune reaction were revealed in immunohistochemical and transcriptomic patterns. Among differentiated elements, several cells were identified expressing typical stem cell markers of embryonic, hematopoietic, neural and mesenchymal lineages in significantly higher number and specific topographic distribution in respect to control valves. Following the longest follow-up ever realized in preclinical models, non-preconditioned decellularized allogeneic valves offer suitable microenvironment for *in vivo* cell homing and tissue remodeling. Manufactured with simple, timesaving and cost-effective procedures, these promising valve replacements hold promise to become an effective alternative, especially for pediatric patients.

## Introduction

Bioprosthetic and mechanical substitutes are currently unique therapeutic options for the rising concern of heart valve disease, affording however time-limited or unsatisfactory biocompatibility [1].

Implanted bioprostheses degenerate following calcific nucleation on cell remnants, interesting fragmented collagen/elastin fibers and/or depending on the mineralization potential of glutaraldehyde (GA) residues [2]–[4]. GA treatment stabilizes extracellular matrix (ECM) against chemical/enzymatic degradation and shields xenogeneic cell epitopes in porcine or bovine bioprostheses [5]. Though, GA cytotoxicity hinders cell viability in valve tissues [6]. Additionally, *in vivo* tissue fatigue may induce GA-coating microfenestrations with xenoantigen exposure and increasing titers of specific IgGs/IgMs early after implantation [7], [8].

During the last decades, several tissue-engineered heart valves (TEHVs) have been tested as alternative surgical solutions to non-viable bioprostheses. Naturally endowed with a tridimensional ECM architecture for satisfactory heart valve physiology, decellularized native tissues are used as scaffolds to be cell-seeded and stimulated in bioreactors for the achievement of mature bioengineered replacements [1]. However, the manufacturing phases for their production are arduous to control and time-consuming.

The exceptional TEHV approach of guided tissue regeneration sidesteps *in vitro* conditionings, conceiving the body as physiological bioreactor: positive hemodynamic evaluations of such a regenerative concept have been already reported in sheep models within short/mid-term follow-ups and recently in the clinics with no mortality up to 6 years [9]–[12].

So far, only two decellularized, GA-devoid porcine matrices have been evaluated in patients with great expectation on post-implantation tissue remodelling. Both applied for RVOT reconstruction, Synergraft valves were implanted in 4 children in 2001 with catastrophic early-term failure, while controversial immunological findings were disclosed for Matrix P ones, used since 2002 in nearly 200 subjects [13]–[17].

Residual xenoantigens in incompletely decellularized xenogeneic grafts might be responsible for hyperacute rejection [13]. In novel TEHVs, it will be mandatory to assess retained biohazardous agents, such as alpha-gal, through specific assays.

TRICOL treatment combines osmotic shock, Triton X100/Sodium Cholate detergents and endonucleases, resulting in complete tissue decellularization, alpha-gal xenoantigens removal and preservation of original scaffold architecture and valve performance [18]–[22].

Following these preliminary *in vitro* validations, TRICOL-treated aortic valves were implanted in Vietnamese pigs (VPs) as allogeneic RVOT replacements with favourable haemodynamic assessments up to 15 months [23].

The present study focuses on the *ex vivo* evaluation of valve re-colonization and demonstrates effective tissue regeneration of decellularized heart valve scaffolds by recipient’s cells after this long-term follow-up.

## Materials and Methods

### 1. Preparation of Decellularized Valve Scaffolds

Aortic heart valves were isolated from 3–4 months old common breeding piglets and submitted to TRICOL decellularization treatment [18]–[23]. Briefly, hearts were obtained in local abattoirs and maintained in antibiotics/antimycotic-complemented cold saline solution until further processing (100 U/mL penicillin, 100 mg/mL streptomycin and 250 mg/mL amphotericin B). After isolation in aseptic conditions, aortic valvulated conduits were decellularized by alternated treatment with hypo- or hypertonic solutions, Triton X100 and sodium cholate detergents, followed by incubation with the aspecific endonuclease Benzonase (Novagen, EMD Millipore, San Diego, CA) for complete removal of nuclear remnants. Effective decellularization was assessed by classic histology, DNA biochemical quantification and nuclear counterstaining with Hoechst (Hoechst 33258; Sigma) as elsewhere reported [23]. The coronary arteries originating from the aortic root were closed through suture and their ostia were trimmed in order to use the allogeneic conduit as pulmonary valve substitute. Further disinfecting treatment was performed before valve implantation by four 24-hours cycles with fresh antibiotic-antimycotic cocktail as elsewhere described [19].

### 2. *In vivo* Implantation and *Ex vivo* Cell and Tissue Analyses on Grafted Heart Valves

The Italian Ministry of Health (IACUC equivalent) authorized the University of Padua to use VPs for the experimental purposes of this study (27/08 C16 project), conforming to *D. Lgs*. 116 (art. 12; 27.01.1992) and to ISO 10993-2 principles. A total of 20 VPs, provided by CISRA Institute (120TO025-ASL 3 Collegno, Torino, Italy), were housed in groups and after their arrival, at least one week was left for acclimation.

General welfare of VPs was assessed daily and was considered satisfactory if body condition score, social, alimentary and locomotory behavior were found normal.

If any clinical (i.e. dyspnea, depression, anorexia, weight loss) or behavioral (i.e. separation from the social group, lack of normal curiosity) abnormality was observed, a complete clinical and cardiological evaluation was performed. In case of severe illness or heart failure, pre-term euthanasia was induced.

Five out of 20 animals were not included in the study due to intra-operative death caused by ventricular fibrillation or arterial embolization, iatrogenic complications after surgery or post-intervention endocarditis. TRICOL-decellularized, allogeneic aortic valves were used for RVOT reconstruction in 11 animals (group A; n = 5 and n = 6 for 6 and 15 months respectively). In sham VPs (n = 2 for each considered evaluation time), the pulmonary valves were resected and re-implanted in the same position. More detailed information about animal husbandry, clinical management, valve implantation and instrumental follow-up have been reported elsewhere [23].

At any experimental sacrifice time foreseen by the study design, each VP was sedated with midazolam (0.3 mg/kg), medetomidine (15 µg/kg) and ketamine (10 mg/kg), administered into the neck muscles, using a 3.5 inch spinal needle connected to a syringe by the means of an extension line (1×2.5×1500 mm) (Prolex extension line, Emiltek srl, Mirandola, Italy). Anaesthesia was induced with isoflurane in oxygen via facemask. Before tracheal intubation, the larynx was desensitized with one dose (1 ml) of 2% lidocaine. Anesthesia was maintained with isoflurane in oxygen, administered via a circular breathing system and alfentanil 20 µg Kg^−1^ was administered as a loading dose, followed by 1–1.5 µg Kg^−1 ^min^−1^ as constant rate infusion. Once thoracotomy was accomplished, each VP was suppressed by an intravenous injection of a mixture of embutramide, mebenzonium iodide and tetracaine hydrochloride (Tanax, Intervet Italia, Segrate, Italy) at 0.3 ml per Kg of body weight, just before explanting engrafted valves.

In order to evaluate the occurrence of *in vivo* regeneration, each TRICOL aortic graft, used to reconstruct the RVOT in Group A, was harvested at 6 or 15 months under sterile conditions together with the proximal and distal anastomosed recipient’s tissues (RVOT muscle portion and native pulmonary artery segment). A longitudinal cut at the level of the cusp commissures was performed to divide the explanted graft in three sectors, each comprising the corresponding leaflet, the Valsalva sinus, the allograft wall and the distal anastomoses. Re-implanted pulmonary roots in Group B were isolated and subdivided as above-mentioned.

#### 2.1 Histology and immunohistochemistry

Histological analyses were performed on thin paraffin sections by standard Haematoxylin and Eosin and Masson’s staining, Alcyan-PAS for glycosaminoglycan detection (GAGs), Heidenhain’s Azan trichrome to analyze collagen fibres, elastic van Gieson for the evaluation of elastic fibres and von Kossa (Sigma) to investigate the presence of calcifications. Observations were accomplished using an Olympus microscope BX51 (Olympus Corporation, Tokyo, Japan).

For immunolocalization experiments, tissues underwent OCT inclusion, snap-freezing and further serial cryostat sectioning (8–9 µm-thick sections). For immunohistochemical experiments using paraffin-embedded tissue samples, deparaffinised thin sections (4–5 µm) were subjected to heat-induced antigen retrieval performed according to standard protocols. A pre-treatment in citrate buffer using a Rapid Microwave Histoprocessor (RHS Milestone, Sorisole, Italy), followed by washes in phosphate buffer and 20 min-incubation in PBS containing 2% milk powder and 0,2% Triton X-100 was carried out.

The following primary antibodies were used:

- Pig CD45 (CD45; AbD Serotec, Oxon, UK), Pig T-cell receptor δ (VMRD Inc., Pullman, IL), B-cells (Abcam, Cambridge, UK), CD68 (Dako, Dakopatts, Denmark), IL-10 (Abcam), Natural Killers (NK; Abcam) and Mast Cell (Abcam) for inflammatory/immune responses;
- Osteocalcin (OC; Abcam) for calcifying cells;
- Von Willebrand factor (vWf; Dako) and CD31 (Santa Cruz Biotechnology, Santa Cruz, CA) for endothelial cells (ECs);
- Smooth muscle actin (SMA; Sigma, St. Louis, MO), osteopontin (OPN; Abcam), vimentin (Dako), type A non-muscle myosin heavy chains (MyHCApla1; [24]), smoothelin (Abcam), calponin (Sigma) and smooth muscle myosin (SM-MyHC; [24]) for interstitial valve cells (VICs) and smooth muscle cells (SMCs);
- Pro-collagen I (Developmental Studies Hybridoma Bank, Iowa City, IA), collagen I (Sigma) and elastin (Sigma) for ECM;
- SSEA4 (Chemicon, Temecula, CA) and OCT4 (Santa Cruz) for embryonic stem cells;
- CD34 (Dako) and CD117 (Santa Cruz) for haematopoietic stem cells;
- CD29 (VMRD Inc.), CD90 (Cymbus, Chandlers Ford, UK) and CD105 (Abcam) for mesenchymal stem cells;
- Nerve Growth Factor receptor (NGFr, Abcam), Nestin (Abcam) and Glial fibrillary acidic protein (GFAP; Chemicon) and Protein Gene Product 9.5 (PGP 9.5; UltraClone Limited, Wellow Isle of Wight, UK) for neural stem cells and neuronal cells.

Primary antibody binding was revealed through HRP-conjugated anti-mouse and anti-rabbit IgGs (Dako) as secondary antibodies. Controls were incubated with mouse or rabbit non-immune IgGs (respectively Sigma and Dako) instead of the primary antibodies. The 3-amino-9-ethylcarbazole (AEC; Sigma) represented the substrate used for revelation. Then, nuclear counterstaining was carried out with Harris’s haematoxylin (Sigma).

A Leica light microscope (Leica, Wetzlar, Germany), connected to a Leica DC300 digital camera, was used for antigen distribution evaluation and image recording. Before photographic acquisition, four microscopic fields (ROI = 0,0374 mm^2^) were selected as representative of the analyzed sample. For each considered antigen, equivalent areas of interest were examined. Antigen distribution was computed as mean percentage ± deviation standard using ImageJ software (http://rsbweb.nih.gov/ij).

#### 2.2 Primary cell cultures

Explanted graft wall specimens and corresponding leaflets were sterile kept at +4°C until further processing. Adventitial, medial and valve interstitial primary cells were obtained by pre-digested explant culture, as described elsewhere [25]. *Adventitia* was carefully stripped from the vessel, minced and cultured in complete medium containing α-MEM (Sigma), 20% FBS (Gibco, Carlsbad, CA), 1% L-Glutamine and 1% Penicillin- Streptomycin (Sigma). Leaflet surfaces and vessel lumen were scraped to remove the endothelium, minced in fragments of about 2–3 mm^3^, incubated with a sterile-filtered digestion solution composed of collagenase I (125 units/ml; Sigma), elastase (8 units/ml; Sigma) and soybean trypsin inhibitor (0,375 mg/ml; Sigma) for 30 minutes at 37°C. Fragments were collected in an OPTILUX non-tissue culture Petri dish (Falcon BD Bioscience, San Diego, CA) and cultured in the complete medium described before. Cells spread from pre-digested fragments after 7–10 days.

Morphological aspects of different primary cultures were observed with a phase contrast Leica DM IRB microscope connected to a Canon Power shot S40 camera.

After first passaging, cells were plated onto coverslips and analyzed immunohistochemically after fixation in 2% p-formaldehyde in PBS pH 7.2. A double indirect immunofluorescence was performed on p2 different cell types by incubating with anti-SMA, FITC-conjugated (clone 1A4; Sigma), and the following antibodies directed versus CD45, MyHCApla1, smoothelin, calponin, SM-MyHC, OPN, OC, SSEA4, OCT4, CD29 and CD90. Alexa Fluor 594-conjugated goat Fab’ to mouse IgGs (Molecular Probes, Carlsbad, CA) and anti-rabbit TRITC-conjugated goat IgGs (Sigma) were used as secondary antibodies. Cell nuclei were identified by Hoechst staining.

Antigen distribution was studied in four microscopic fields (corresponding to 0,0374 mm^2^) using a Zeiss Axioplan epifluorescence microscope (Zeiss MicroImaging GmbH, Göttingen, Germany) and images were acquired using a Leica DC300F digital camera. ImageJ software was used to quantify cell expression, further elaborated as mean percentage ± deviation standard.

#### 2.3 Cell survival

Cell mitotic events were studied with a specific marker of cell proliferation, i.e. Phospho-Histone 3 (PH3; Ser 10 Upstate, Lake Placid, NY). Antibody binding was indirectly revealed with an anti-rabbit HRP-conjugated secondary antibody and AEC as substrate or, for quantification experiments, using anti-rabbit TRITC-conjugated goat IgGs. Haematoxylin or Hoechst were used to counterstain nuclei.

A TUNEL-based in situ detection kit was applied to evaluate DNA fragmentation in apoptotic cells (ApopTag Plus Fluorescein/Peroxidase In Situ Apoptosis Detection Kits; Chemicon) following the manufacturer’s instructions.

Observations and photographic recordings were collected by means of Zeiss Axioplan epifluorescence microscope or AxioImager photomicroscope (Zeiss). Quantification of positive cell events in four microscopic fields (corresponding to 0,0374 mm^2^) was obtained by means of ImageJ software and expressed as mean percentage ± deviation standard.

#### 2.4 Scanning Electron Microscopy (SEM)

Specimens were fixed with 2,5% glutaraldehyde and 2,5% formaldehyde in 0,1 M phosphate buffer. After progressive dehydration with ethanol solutions from 20% to 100%, they were critical point dried and vacuum metalized. A Jeol JSM 6490 microscope (Jeol, Peabody, MA) was utilized for observation and recording. Images were elaborated by Photoshop CS2 software to ease the visualization of different ECM components and cell elements: basal lamina/ECM, ECs, endothelial microvilli and red cells were colored in green, blue, purple and red respectively.

#### 2.5 Transmission Electron Microscopy (TEM)

Tissues, fixed with 2,5% glutaraldehyde and 2,5% formaldehyde in 0,1 M phosphate buffer, were rinsed with the same buffer, treated with 2% osmium tetraoxide (Agar Scientific Ltd, Essex, UK), dehydrated in graded ethanol solutions and embedded in Araldite/Epon. Semithin sections were conventionally stained with 1% toluidine blue. Thin sections were collected on formvar-coated 2×1-mm-slot copper grids and contrasted with uranyl acetate and lead citrate. Observations and photographic records were realized using a Philips CM12/STEM electron microscope (Philips, Eindhoven, The Netherlands).

#### 2.6 Microarray analyses

Tissue samples were stored in RNAlater (Ambion, Austin, TX) until RNA extraction. Total RNA and small RNAs were extracted independently from each tissue sample with TRIzol reagent (Invitrogen, Carlsbad, CA) in association with PureLink miRNA isolation kit (Invitrogen) and flashPAGE fractionator system (Ambion). Briefly, a tissue amount of approximately 200 mg was homogenized in 3,5 ml of TRIzol with a tissue homogenizer (IKA Werke, Staufen, Germany). After chloroform addition and centrifugation, the colorless upper aqueous RNA containing phase was mixed with 96–100% ethanol to a final concentration of 35% ethanol and loaded to PureLink membrane to separate total and small RNAs following the manufacturer’s instructions. Total and small RNAs were quantized using Nanodrop ND 1000 spectrophotometer (Thermo Fisher Scientific, Waltham, MA). Total RNA samples were tested for quality on Agilent Bioanalyzer 2100 using RNA 6000 Nano LabChip (Agilent, Santa Clara, CA): an average value of 7 as RNA Integrity Number (RIN) was set to proceed in further analyses. MicroRNAs (MiRNAs or MiRs) were purified from small RNAs through flashPAGE after testing for their presence with the 2100 Small RNA (Agilent).

For Messenger RNA gene expression, Ensembl transcripts (Ver. 56) and UniGene (Ver. 38) pig sequences were used to develop a dedicated microarray platform (90 K CombiMatrix, Irvine, CA) in order to monitor mRNA expression (GEO ID: GPL13259). A total RNA quantity of 1 µg was linearly amplified and labelled with the addition of biotinylated nucleotides according to the Ambion MessageAmp II aRNA Amplification kit (Ambion). Biotinylated UTPs were incorporated into the aRNA during the *in vitro* transcription reaction. Following purification, 18 µg of aRNA was fragmented using the Ambion Fragmentation Kit (Ambion). Intact and fragmented aRNAs were tested on Agilent Bioanalyzer 2100 using RNA 6000 Nano LabChip. Fragmented aRNA was hybridized to pre-hybridized 90 K Combimatrix microarrays.

Images of hybridized microarray were analyzed for fluorescence signal quantification using the Combimatrix imaging software. Negative control probes were introduced in the microarrays and used to calculate the background value (filter). A Gene Ontology (GO) analysis was performed using FunNet transcriptional network analysis [26], [27].

For evaluation of miRNA expression, dedicated microarrays composed of specific probes for miRNAs and associated background probes (GEO ID: GPL13322) were developed. 150 ng of miRNA were hybridized to microarray platform to perform RAKE experiments in duplicates. Quantization of miRNA concentration was based on a specific titration curve obtained from the signals of spike RNA introduced in the reaction mixture. A regulatory network was produced also according to gene interactions retrieved from STRING database [28] and visualized through Cytoscape [29].

All microarray data have been deposited at GEO database with accession number GSE27853.

### 3. Statistical analysis

All data regarding the protein expression analyses are expressed as means ± SD. Statistical differences between the allografted aortic tissues and the repositioned pulmonary valves were determined using Student's paired *t*-tests. Statistical significance was considered for p<0.05. For gene expression investigation, raw data obtained from the fluorescence signal quantification of microarrays were normalized with the quantile method. In order to identify differential expression, median FDRs of 0,17% and 0% were considered for the SAM analysis of respectively mRNA and miRNAs. Analyses on miRNAs and mRNAs were integrated in relation to Pearson correlation gene expression.

## Results

### 1. *Ex vivo* Assessments on Explanted Heart Valves

#### 1.1 Macroscopic inspection

**Figure 1:**
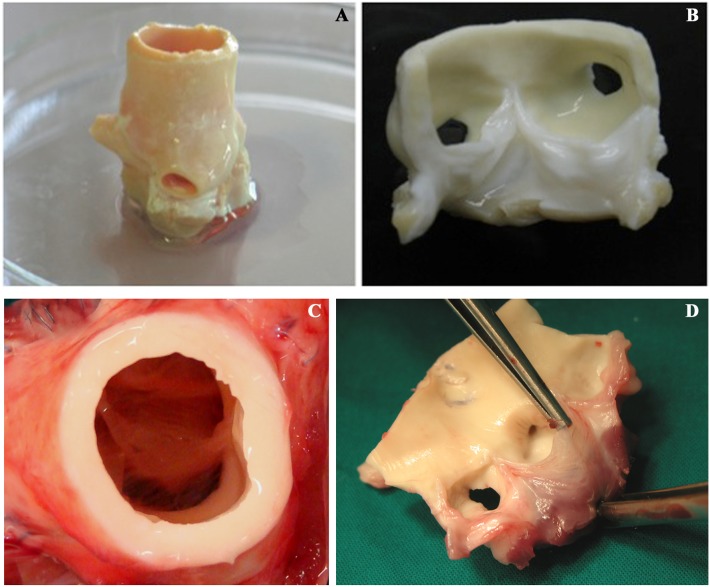
Macroscopic appearance of TRICOL allogeneic aortic valve before and after implantation. Allogeneic substitutes demonstrated similar gross morpho-anatomic structure to native valves without signs of leaflet fenestration, rupture or degeneration both after decellularization (C–D) and explant at 15 months (C–D).

Prior to implantation, TRICOL aortic valvulated conduits exhibited patency and normal morpho-anatomical characteristics (Figure 1A–B). All allografted VPs (n = 11, Group A) demonstrated adequate hemodynamic performance and proper implant growth, as sham animals (n = 4, Group B) [23]. Explanted allografts showed normal appearance without thrombi, vegetation, rupture or RV hypertrophy/aneurysmatic dilatation (Figure 1C–D). Group A cusps were soft, translucent and free from degenerations as those from Group B, except two specimens (6 and 15 post-implantation months, respectively) with mild thickening and sclerosis.

Calcification was detected at suture level in both experimental groups.

#### 1.2 Histological and immunophenotypical studies

**Figure 2:**
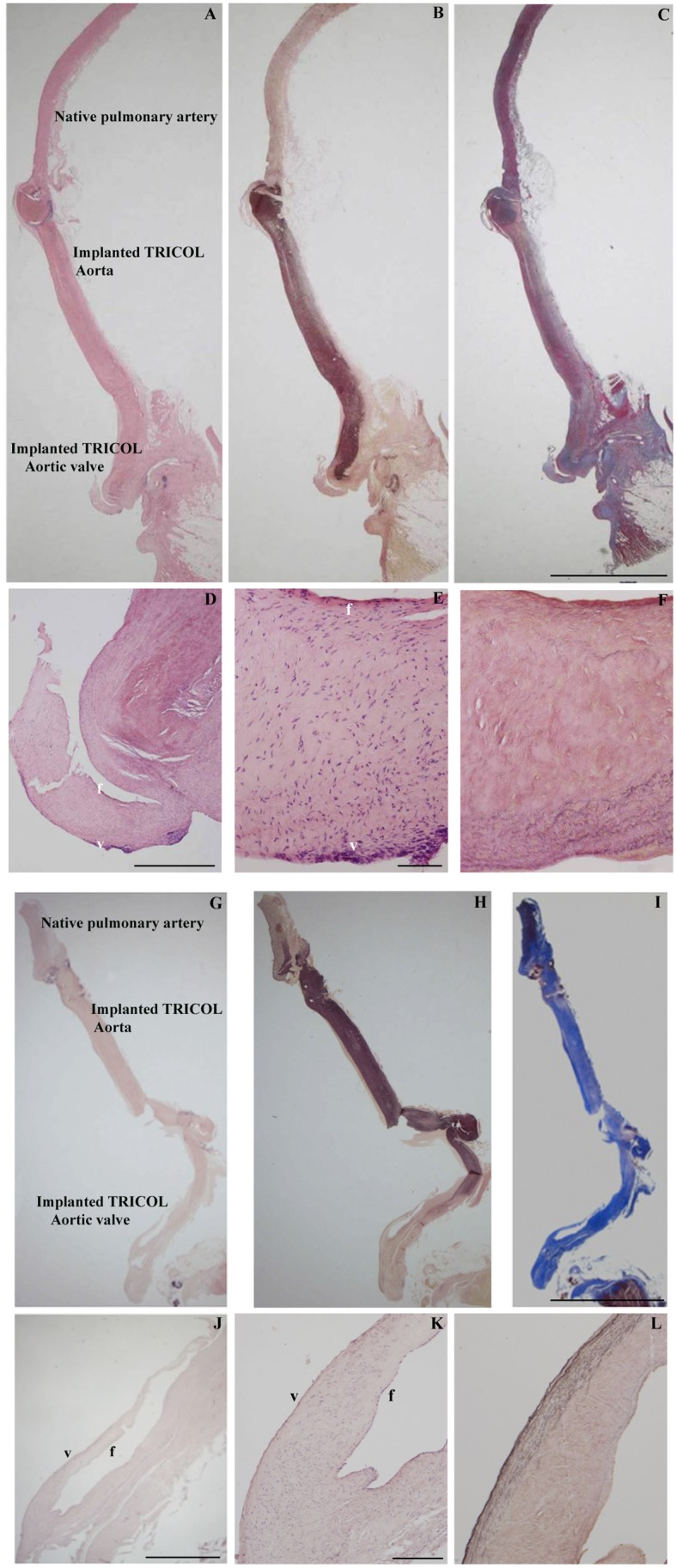
Histologic evaluation of explanted allografts. Panoramic (A and G: H&E, B and H: Elastic van-Gieson, C and I: Azan’s Heidenhain trichrome, magnification: 5 cm) and close-up (D–F and J–L) allograft views respectively at 6 and 15 months after surgery: note trilaminated arrangement with recipient’s repopulating cells on both leaflet sides, i.e. *ventricularis* (v) and *fibrosa* (f) (D–E, J–K: H? F and L: Elastic van-Gieson). Magnifications: (A–C and G–I) 1 cm; (D) 500 µm; (E, F) 100 µm; (J) 700 µm; (K, L) 200 µm.

In allograft walls, histomorphological analyses, i.e. Heidenhain’s Azan trichrome, elastic van Gieson and Alcian-PAS, revealed appropriate ECM structure without rejection and/or degeneration signs (e.g. elastic fiber fragmentation, fibrosis) or evidence of increased interfibrillar space (Figure 2A–F and 2G–L respectively for 6 and 15 months after implantation).

Focal dystrophic calcifications, i.e. single cells positive for von Kossa and OC, interested anastomotic areas between recipient’s pulmonary artery and donor’s decellularized aorta (Table S1 and Figure S1 A, B and C for the immunophenotypical quantification of cells populating respectively the *intima*, *media* and *adventitia*).

**Figure 3:**
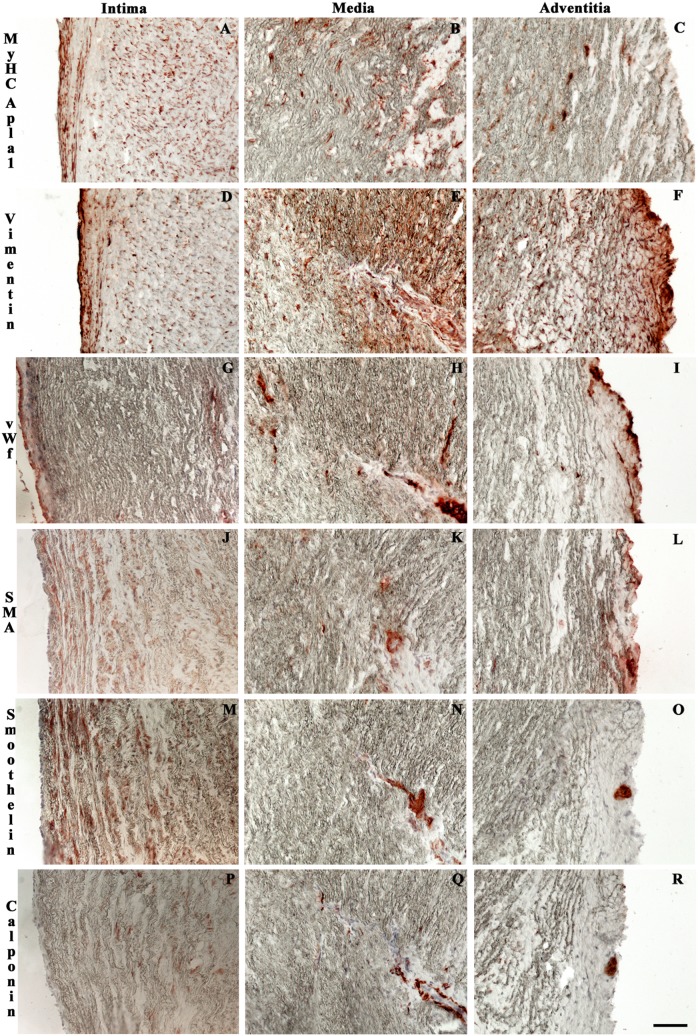
Immunohistochemical characterization of allograft walls at 15 months. Vimentin- and MyHC_Apla1_-positive fibroblasts (A–F) with broad SMA, rare smoothelin and calponin detection (G–O). Re-endothelialization was observed at the intimal layer (G). Novel mature arterioles and capillaries were identified in *adventitia* and *media* (H, I, K, L, N, O, Q and R). Magnification: 100 µm.

The allograft intimal layer was covered by a dense endothelial lining, similarly to repositioned RVOTs (p = NS; Figures 3G and S1A). In the endothelium layer, some myoendothelial elements could be recognized for the expression of vWF and SMA.

Activated fibroblasts, positive for MyHCApla1, vimentin, OPN and SMA, spread extensively in the *media* (p<0,05 in respect to group B; Figures 3A–F, 3J–L and S1B). In comparison to sham animals, fewer smoothelin-, calponin- and/or SM-MyHC-expressing SMCs were found, mostly populating neovascularized medial regions (p<0,05; Figures 3M–R and S1B).

Already after 6 months, a rich medial capillary network was present and at last explanttime, both *media* and *adventitia* displayed newly re-formed, mature *vasa vasorum* (vWF-expressing endothelial lumen and SMA-, smoothelin- and calponin-positive arteriolar wall) (Figures 3H–I, K–L, N–O and Q-R, as well as S1B and C).

Procollagen-I synthesis was detected in cells colonizing the allograft, more than in those populating the autograft (p<0,05; Figure S1A, B and C). A progressive decrease in expression of CD29, CD34, CD105, SSEA4 and OCT4 was observed during time in both groups, with a significant permanence of stem cell epitopes, mainly of mesenchymal, embryonic and neural lineages, in the 15-month allografted wall (p<0,05; Figure S1A, B and C).

In Group A, a slight intimal thickening could be observed.

**Figure 4:**
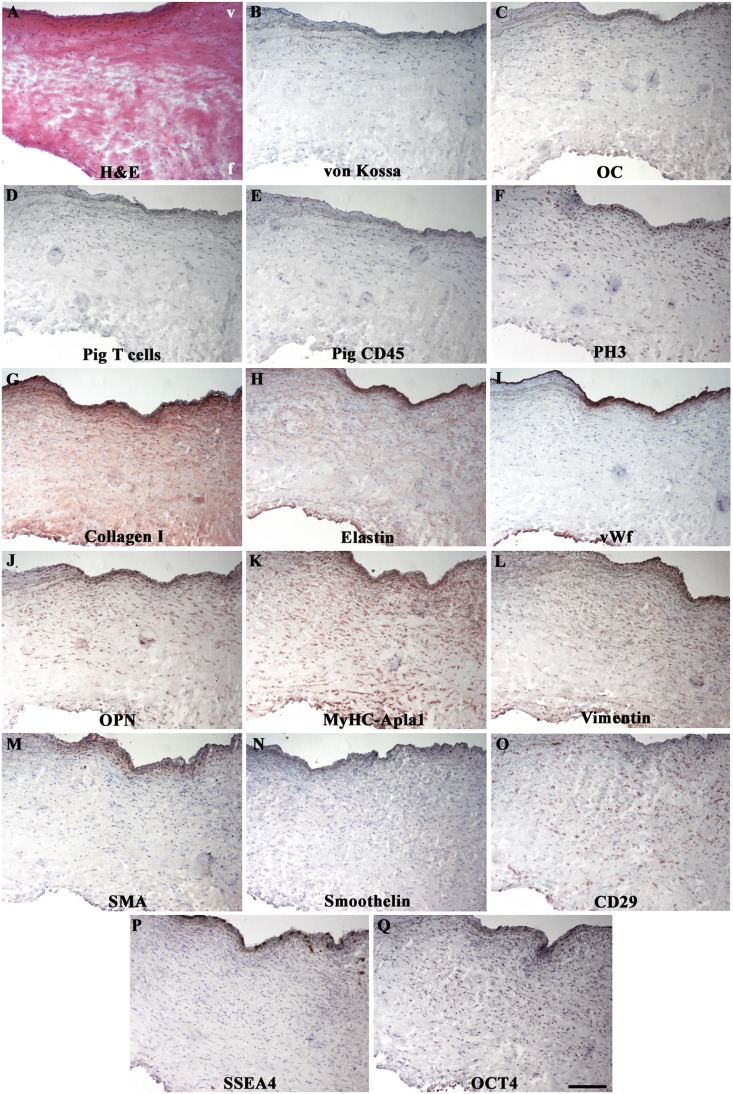
Immunohistochemical profile of recellularized leaflets at 6 months. Undetectable calcifications (B, C) or immune rejections against allogeneic tissues (D, E). Conserved trilaminated ECM architecture (A, G and H). Native-like EC (I) and VIC phenotypes (J–N). Stem cell markers of mesenchymal (O) and embryonic (P and Q) lineages, mainly expressed in *ventricularis*. In (F), PH3-positive leaflet-colonizing cells. V = *ventricularis*; f = *fibrosa*. Magnification: 200 µm.

In allograft cusps, the original trilaminated structure was preserved with intact elastic *lamellae* in *ventricularis* and regular collagen and GAG distributions in *spongiosa* and *fibrosa* (Figures 2F–H and J–L, 4A, G and H). No calcifications were apparent (Figure 4B, C) and inflammatory infiltration could be revealed only by minimal CD45 immunodetection (Figure 4D, E), similarly detected in sham tissues (p = NS; Figure S1D).

Most cusps were completely re-endothelialized (Figure 4I), apart for 3 non-coronary cases, one after 6 and two after 15 months, with partial coverage and platelet attachment.

Fibroblast migration patterns interested leaflet base towards free middle and distal portions. In one case at 6 implantation months and in most samples at the last observation time, the *interstitium* was repopulated by many OPN, MyHCApla1, vimentin, SMA and smoothelin-positive cells with analogous distribution to a native leaflet (Figure 4J–N; Figure S1D; for comparison to a native tissue, see [25]). In respect to B group, recellularized regions showed higher expressions of embryonic (p<0,05), mesenchymal (mainly p = NS) and neural (p = NS) stem cell markers, with furthermost topographic location in the *ventricularis* layer (Figures 4O–Q and S1D).

In comparison to group A, autografts displayed a lower number of CD68-, OPN- and IL-10-positive cells (often p<0,05; Figure S1D), known as M2 macrophages possessing anti-inflammatory and reparative properties. At 15 months, repositioned RVOTs exhibited good cellular and ECM preservation with undetectable degenerations (Figure S1D and Table S1 for a detailed statistical comparison between groups A and B).

#### 1.3 Allograft primary cell cultures

**Figure 5:**
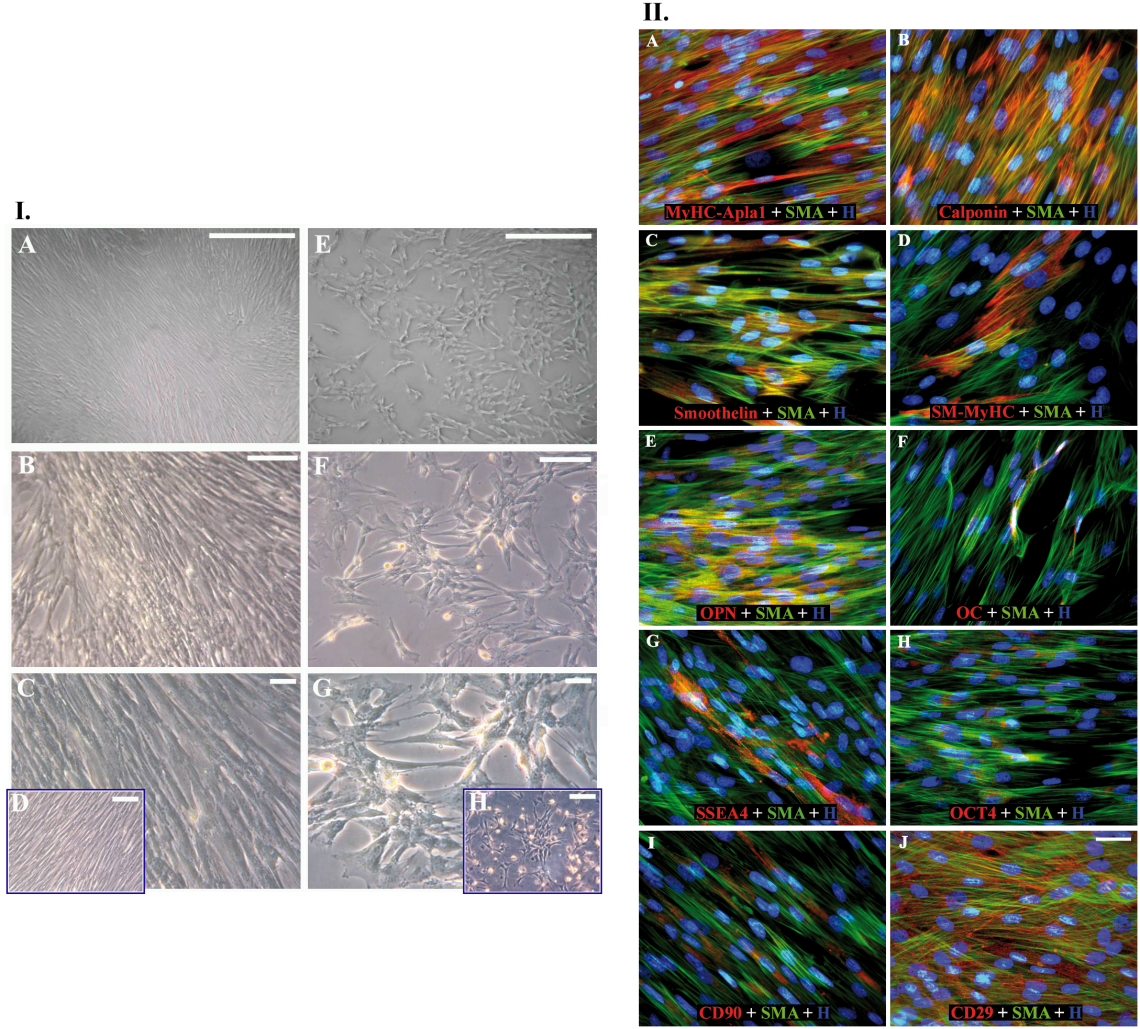
Morphological and immunophenotypical analyses of primary cultures from 15-month regenerated allogeneic walls. I. Two main morphologies could be observed during culturing medial cells: a cell line more elongated reaching confluence with hill and valley distribution (A, B and C) and another one characterized by spindle-shaped cells growing in clusters (E, F and G). Such morphological aspects revealed a fine analogy of these two primary cells respectively to mature artery SMCs (D) and bone marrow mesenchymal stem cells (H), both of porcine origin. Magnifications: (A, E) 150 µm, (B, F) 100 µm, (C, D, G, H) 50 µm. II. Immunophenotype of primary cultures from 15-month regenerated allogeneic walls. Wide FITC-conjugated SMA-positivity (green), less usual co-immunodetection with smoothelin or SM-MyHC (in red) (A–E), rare OC positivity (F), extensive CD29 expression (J) in contrast to SSEA4, OCT4 and CD90 (G–I) (in green). Magnification: 30 µm.

Primary cultures (*adventitiae*, *mediae* and cusps) revealed two main morphological phenotypes (respectively, Figure 5.I.A–C and E–G) similar to SMCs or bone marrow-mesenchymal stem cells (Figure 5.I.D and H respectively). Most *media*-extracted cells were positive for SMA, MyHCApla1 and OPN, while modestly expressing smoothelin, calponin and SM-MyHC (Figure 5.II.A–E, Table 1 for quantitative description). Absence of porcine CD45-leukocytes and rare OC detection were remarkable (Figure 5.II.F, Table 1). Some myofibroblasts denoted immature phenotype for SSEA4, OCT4, CD90 and predominantly CD29 expression (Figure 5.II.G–J, Table 1).

**Table 1:** Immunophenotype of primary cultures derived from explanted valve allografts.

Antigens	Medial cells (%)	Adventitial cells (%)	VICs (%)
Pig CD45	ND	0,00±0,01	0,00±0,01
MyHC-Apla1	82,51±5,30	87,06±8,21	95,26±1,93
Calponin	26,57±3,69	11,30±7,88	7,11±0,97
SM-MyHC	28,15±9,01	29,71±13,61	5,02±2,96
Smoothelin	31,03±7,16	12,56±6,15	3,41±2,00
OPN	68,06±16,33	63,41±13,28	88,91±7,02
OC	1,25±0,18	1,06±0,05	0,98±0,23
SSEA4	15,89±4,02	7,41±5,38	20,26±4,59
OCT4	7,06±6,99	10,06±4,45	29,97±1,81
CD90	3,10±2,01	1,15±0,86	25,80±2,22
CD29	74,36±4,13	52,29±22,78	88,30±11,01

Legend: Percentage of antigen expression after normalization to counted nuclei. ND: not detectable.

Primary cultures extracted from explanted tissues at 15 months were characterized immunophenotypically for markers commonly expressed in inflammation (CD45), SMCs and/or VICs (OPN, MyHC-Apla1, Calponin, SM-MyHC and smoothelin), calcification (OC) and stemness (SSEA4, OCT4, CD90 and CD29).

#### 1.4 Cell survival aspects

After 15 follow-up months, engrafted cells were highly proliferating with about 53,69±17,18% phospho-histone 3 (PH3)-positive cells in entire regenerated walls, averaging single percentages of 45,18±16,16% in *intima*, 43,56±24,65% in *media* and 72,33±11,42% in *adventitia* (Figure S2A–C). An intense mitotic activity interested allograft cusps too (57,32±10,31%) (Figure 4F).

Similarly to native valves, repopulated allografts rarely exhibited TUNEL-positivity, i.e. 3,92±2,57% cusp cells and 3,17±1,31% wall cells (2,37±1,13%, 4,63±1,81% and 2,53±0,98% respectively for *intima*, *media* and *adventitia*). Conversely, native cells in Group B explants presented DNA fragmentation after 6 months, returning to physiological levels at longer observation (not shown).

#### 1.5 SEM ultrastructure

**Figure 6:**
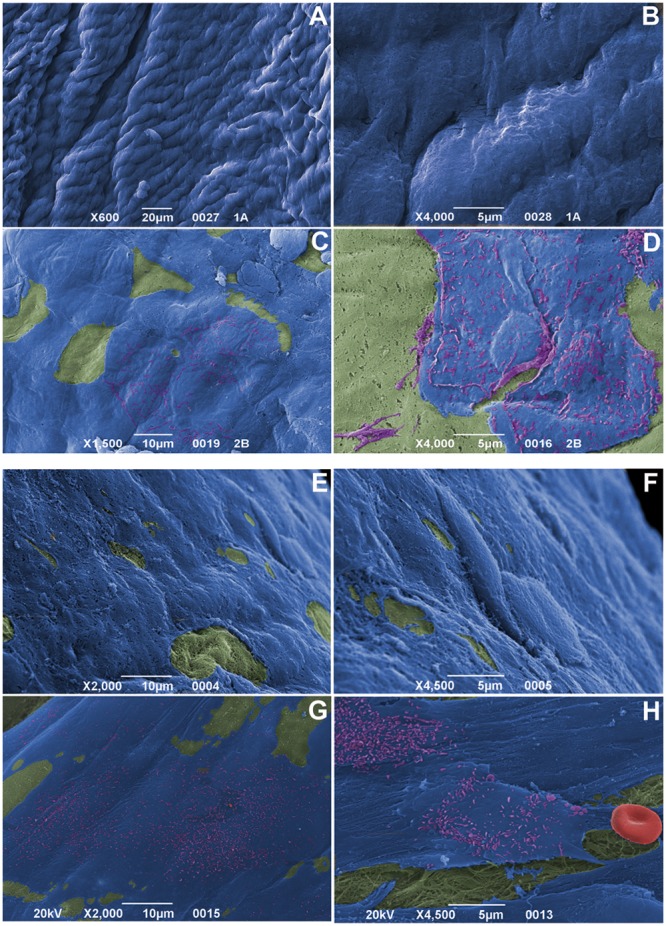
Scanning electron microscopy on allograft cusps at 6 and 15 implantation months. Almost complete endothelial coverage (blue), progressive EC acquisition of surface microvilli (purple) and no platelet aggregation onto *fibrosa* (green; at 6 months in A–B, after 15 months in E–F) and *ventricularis* (green; at 6 months in C–D, after 15 months in G–H). Note the absence of fibrin deposition, as well as no red cell (red) entrapment in H. Magnifications: (A) 20 µm; (B, D, F, H) 5 µm; (C, E, G) 10 µm.

In both observational times, re-endothelialization was nearly complete on leaflet and intimal allograft surfaces with typical microvilli-rich EC phenotype (Figure 6A–D and E-H for specimens at 6 months and 15 months respectively). However, few samples at 6 months showed unusual morphologies (not shown). Superficial fibrin depots or ECM exposure were not detected.

#### 1.6 TEM ultrastructure

**Figure 7:**
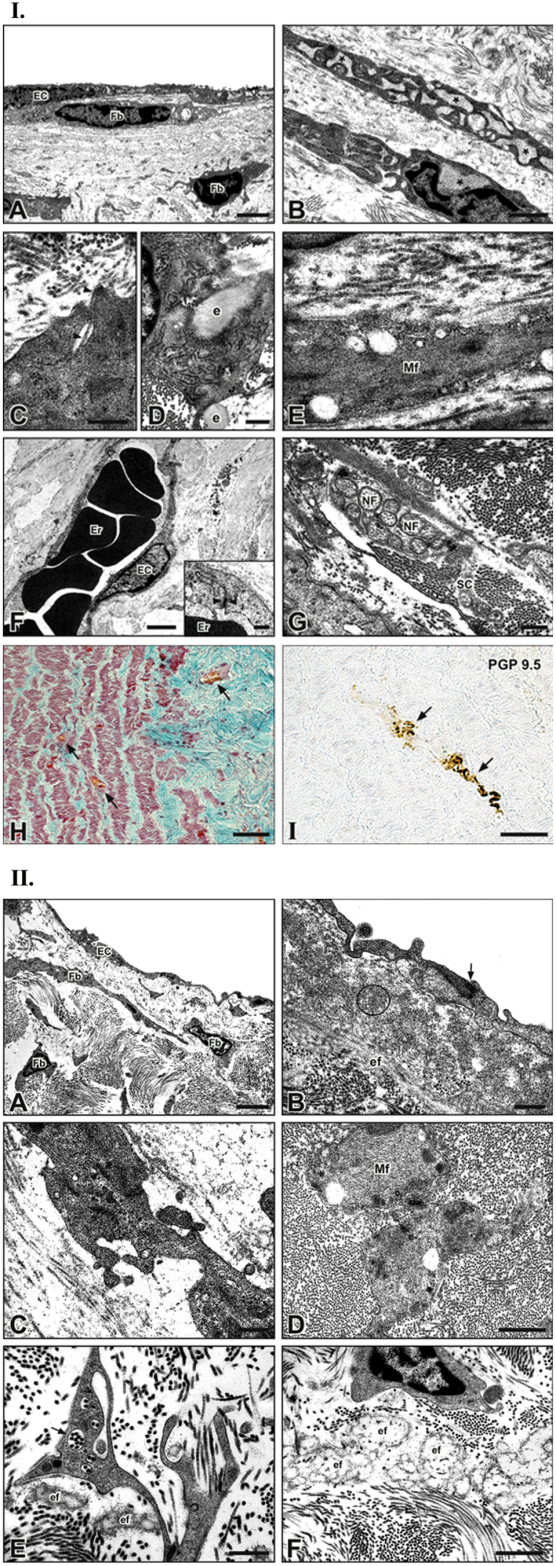
Transmission electron microscopy on allograft walls. ECs covering intimal surface (A); underneath the *intima*, fibroblasts (Fbs) with dilated endoplasmic reticulum (B; asterisks), synthesizing collagen fibrils from fibril-forming channels (C) and elastin fibers (e) (D) or with abundant myofilaments (mf) (E). Erythrocytes (Er)-containing capillaries coated by ECs joined by tight junctions (opposite arrowhead in inset) (F). Amyelinic nerve fibers (NF) encapsulated by Schwann cells (SC) (G). Blood vessels (arrows) in *media* and *adventitia* after trichrome staining (H). Neural marker PGP9.5-expressing nerve fiber (arrows) in *media* (I). Magnifications in I: A 3 µm; B 1 µm; C, D, G 0,5 µm; E, F inset 0,25 µm; F 2 µm; H, I 250 µm. **Transmission electron microscopy on allograft cusps.** ECs onto intimal surface and sub-intimal Fbs (A). Immature intercellular junction (opposite arrowheads) between ECs adhering elauninic fibers (ef) surrounded by fibrillin microfibrils (encircled area) (B). Interstitial myofibroblasts showing intercellular junctions (opposite arrowheads) (C). Interstitial SMCs containing microfilaments (Mf) (D). Fbs with multiple fibril-forming channels (arrow) and adjacent efs (E). Abundant efs in *interstitium* (F). Magnifications in II: A 3 µm; B, C, E 0,5 µm; D, F 1 µm.

Since early follow-up intervals, the luminal surface of allograft walls was covered by an almost continuous monolayer of well-joined, flat ECs (Figure 7.I.A). A 15 µm-thick sub-endothelial fibrillin-rich layer was often detectable.

The *tunica media* was populated by scarcely interconnected cells showing dilated rough endoplasmic reticulum (Figure 7.I.B) and figures of extracellular matrix neosynthesis consisting in irregular indentations, enveloping elaunin fibers and the typical collagen fibril-forming channels (Figure 7.I.C, D).

Spindle-shaped cells, abundant in actin myofilaments, occupied interlaminar spaces (Figure 7.I.E). Neoangiogenic aspects were evident in *media* and *adventitia* due to the presence of capillary vessels, lined by a continuous endothelium endowed with intercellular junctions (Figure 7.I.H, F inset). In these *tunicae*, immunohistochemically detected nerve fibers exhibited the typical features characterizing amyelinic fibers being surrounded in groups by single Schwann cells (Figure 7.I.G and I).

Allograft cusp surfaces were covered by a continuous monolayer of flattened endothelial-like cells anchored to a thin basal lamina and showing immature tight junctions (Figure 7.II.A, B). An additional 3–4 µm-thick layer of elastin fibers/fibrillin microfibrils was often detectable underneath the regenerated endothelium (Figure 7.II.B). Cuboidal ECs, containing prominent Golgi apparatus and dilated rough endoplasmic reticulum, adhered to the luminal surface of two allografts explanted after 6 and 15 months, respectively.

As in native valve leaflets, most cells populating the *interstitium* unveiled fibroblast-like features (Figure 7.II.A, E) and were mixed to others resembling myofibroblasts (Figure 7.II.C) or SMCs (Figure 7.II.D). Most of these cells exhibited lots of collagen fibril-forming channels (Figure 7.II.E) and lied in an ECM enriched by maturing elauninic fibers, surrounded by fibrillin microfibrils (Figure 7.II.E, F).

Nevertheless, some allograft portions were acellular or contained apoptotic/degenerated cells and/or swelled collagen fibrils (not shown).

B grafts showed well-preserved ECM fibrous components, but marked suffering/apoptotic cells after 6 implantation months, with restriction to ECs solely for the longer follow-up (not shown).

#### 1.7 mRNA and miRNA profiles

**Figure 8:**
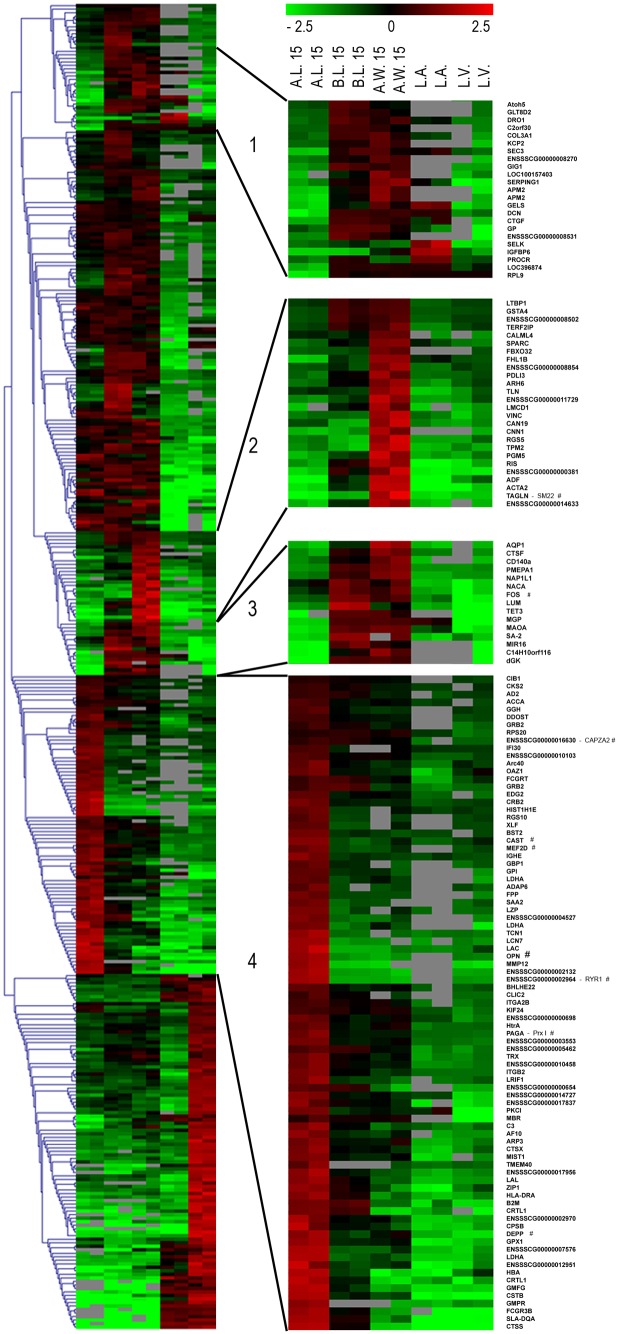
Gene expression profiles at 15 months post-implantation. Heat map of differentially expressed genes: Colors based on logarithmic against averaged gene expressions. Green: down-regulated genes; red: up-regulated ones. A.L.: Allograft Leaflet; B.L.: Autograft Leaflet; A.W.: Allograft wall; L.A.: Left Atrium; L.V.: Left Ventricle.

**Figure 9:**
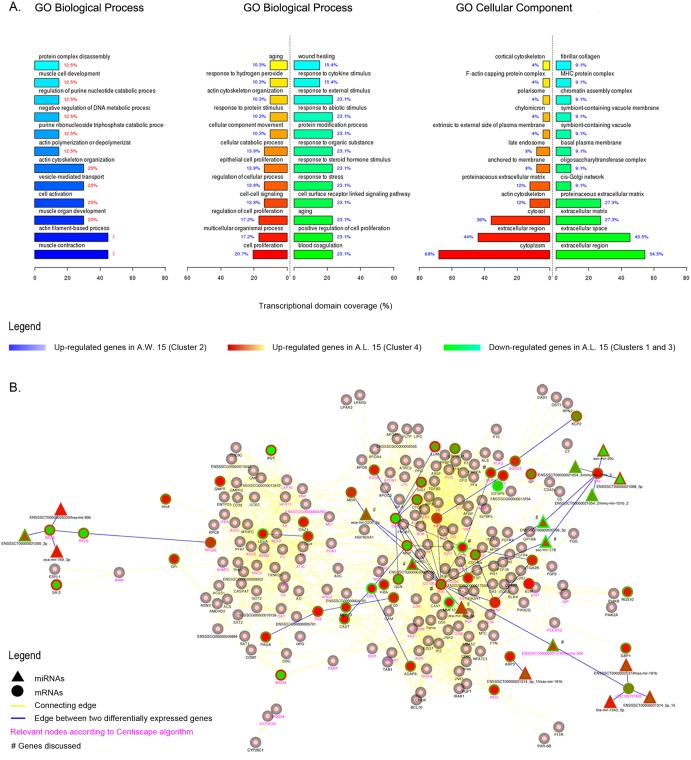
Gene expression profiles at 15 months post-implantation. Histograms representing enriched Gene Ontology terms for clusters 1, 3, 4 (green and red bars) and 2 (blue bars). P-value ≤0,05 (A). Gene interaction network derived from STRING database analysis of up- and down-regulated genes on A.L. tissues (clusters 1, 3, 4). Pink nodes with grey border connect elements between up- or down-regulated nodes in analyzed clusters. Central color node: gene expression in A.L. tissues; border: B.L. tissues. Yellow edges link connected nodes, while blue edges those altered in analyzed clusters. Purple gene names represent relevant nodes according to Centiscape algorithm. *Genes discussed in the text (B).

Genes prevalently expressed in long-term regenerated arterial walls found involvement in ECM binding, smooth muscle contraction and development, as highly activated SM22-alpha (Figures 8 and 9A). Indeed, up-regulation of CAPZA2, CAST, GPX1, MEF2D and RYR1 smooth muscle genes could be observed in allografts’ transcriptome (Figure 8). Gene expression profiles were correlated to immunohistochemical results to confirm neosynthesis of these proteins and reduce eventual stabilizations in the improvement of their half-life, resulting in endorsed genetically activated transcription of ribosomal proteins (Figure 8).

Microarray analyses identified three informative clusters in repopulated allograft leaflets (Figure 8; Table S2). Most up-regulated genes were involved in cell proliferation (20,7%; p-value 2,65E-03) – especially endothelial (13,8%; p-value 7,1E-03) –, cellular catabolic process (13,8%; p-value 2,58E-04), cellular movement/response to protein stimulus (10,3%; p-value 5,84E-03 and 8,83E-03) and response to hydrogen peroxide (10,3%; p-value 3,78E-03) with peroxiredoxin I (PRX I) overexpression (Figure 9A).

Down-regulated genes found involvement in aging (23,1%; p-value 1,11E-03), response to stimuli (23, 1%; p-value 1,04E-03) and wound healing (15,4%; p-value 8,49E-03) (Figure 9A; Table S2).

Interestingly, MHC protein complex class was enriched in down-regulated genes (9,1%; p-value 1,39E-02), indicating scarce or null inflammatory/immune events (Table S2).

Mutual gene interactions and their miRNAs-dependent regulation were considered to identify gene regulatory networks engaged in graft’s cell repopulation (Figure 6). Remarkably, vascular cells-related genes decidual protein induced by progesterone (DEPP) and OPN were up-regulated in recellularized leaflets (Figure 9B). In association to OPN, the most interconnected node in the analysis was FOS, i.e. FOS FBJ murine osteosarcoma viral oncogene homolog gene, regulator of cell proliferation and differentiation (Figure 9B).

Protein synthesis and mRNA stability are extremely influenced by miRNAs, e.g. short RNA sequences important modulators of biological functions. miRNAs, directly FOS-associated, were miR-504, miR-92a, ENSSSCT00000020005, miR-378, miR-220b and ENSSSCT00000020296 (Figure 9B).

## Discussion

The inability of current valve prostheses to grow condemns pediatric patients with congenital heart defects to undergo repeated surgical interventions aimed at restoring cardiac function, but unfavorably raising peri-operative mortality. Additionally, a highly active calcium metabolism can seriously compromise the durability of implanted bioprostheses in younger subjects [30].

The present comprehensive analysis proves the *in vivo* promising self-regeneration abilities of TRICOL allogeneic replacements in 15 follow-up months of hemodynamic and biological performance.

For critical assessment of RVOT reconstruction over time, the VP’s slow somatic growth demonstrates the reliability of such an animal model in simulating a developing juvenile human being [23].

Porcine valve architecture and biomechanical strength are unaffected by TRICOL decellularization [18]–[22], providing eligibility to adequate hemodynamic profile *in vivo*. Noteworthy, sulfated GAG and DNA contents in explants at 15 months are comparable to native conditions [23].

In the present *ex vivo* investigation, histopathological, transcriptomic, ultrastructural and immunophenotypical profiles of implanted decellularized allogeneic heart valves were carefully examined after a long-term TEHV preclinical model. No inflammation, rejection and/or calcification were observed in allograft tissues, apart from mineralization circumscribed only to anastomotic regions in both allografts and autografts. In previous independent echocardiographic analyses, Steinhoff and Hopkins similarly reported evident hyperechogenic areas in subvalvular muscle areas, as pro-inflammatory/pro-calcific effects of the surgical injury itself [9], [11] rather than associated to structural deterioration.

Endothelial molecular pathways were activated on implanted allogeneic tissues, as shown by up-regulated genes involved in EC functions. Outstandingly, OPN, DEPP and FOS share specific expression in ECs, improving their migration, adhesion, proliferation and protection from dysfunction [31]–[33]. As a result of this complex molecular activation, all surfaces exposed to recipient’s blood were re-endothelialized. Allograft *intima* was also inhabited by vWF- and SMA-positive myoendothelial progenitors, early transitional stage of SMC differentiation in arterial embryogenesis [34]. Microarray analyses and immunophenotypical characterization on primary cell cultures of allograft walls confirmed gradual loss of stem cell markers for the progressive acquisition of SM22, inducing filamentous actin bundling, as well as of other contractile smooth muscle proteins, necessary to sustain the differentiated vascular phenotype [35]. Indeed, up-regulated miRNAs, like miR-504, influenced cell cycle, angiogenesis control and functional remodelling of implanted tissues. A dynamic fibroblast engraftment interested over time both cusps and walls, especially the adventitial *tunica*, as assessed by immunohistochemistry on tissues and primary cultures. The high expression of OPN shown by the VIC- and SMC-like cells repopulating the allografts is very similar to that observed in healthy heart valve tissues and likely ensures protective effects against calcific events [25].

Hallmarks of on-going ECM neosynthesis were prominent in leaflet and wall fibroblasts, as evidenced by procollagen I-immunodetection and many, ultrastructurally revealed fibril-forming channels and fibrillin microfibrils-surrounded elaunin fibers.

Since first implantation months, neoangiogenesis/vascularization phenomena, molecularly boosted by up-regulated miRNAs, e.g. miR-92a [36], interested allograft walls with organization in mature capillaries and *vasa vasorum*, well-documented immunohistochemically and ultrastructurally. New blood vessels might have developed from original vasculature’s ECM scaffolding preserved during decellularization, as well as sprouted from *vasa vasorum* supplying the anastomosed recipient’s pulmonary wall. The resulting oxygenation and nutrient provision in implanted matrices is likely to have further improved tissue viability and remodeling, as evidenced by the high survival rate detected in all homed cells. Observed miRNAs and PRX I overexpression correlates to this increased cell survival, indicating that this up-regulation should prevent apoptosis by scavenging reactive oxygen species [37]–[39].

Putative signs of novel vegetative innervation, i.e. immunohistochemically and ultrastructurally identified nerve fibers in *adventitia* and *media*, further indicate potential functionality associated to tissue remodeling. As for neovasculature, re-innervation might have regenerated from preserved ECM of original nerve fibers [40].

Apparently, TRICOL scaffolds are providing a suitable regeneration niche, where recipient’s cells could reside and participate to proper tissue restoration. As further confirmation, many M2-polarized macrophages were found concurring to such regeneration process [41]. The morphological and immunocytochemical characterizations of the primary cultures obtained from explanted tissues, consistent with the transcriptomic results and tissue observations, allow inferring the nature of the engrafting cell sources, i.e. two different phenotypes with high resemblance to mesenchymal stem cells of bone marrow origin and SMCs. Hence, the repopulation phenomenon might have resulted from a systemic bone marrow cell mobilization and a local smooth muscle cell spreading from the adjacent anastomosed pulmonary artery. Actually, the first event is promoted after any surgical intervention: a facilitated diffusion of cytokines and growth factors had likely contributed to the creation of new blood vessels and favored the recruitment/motility of neighboring SMCs [42].

Although engrafted cells acquired a migrating phenotype during observation, some allografts exhibited incomplete re-endothelialization and repopulation. Such conditions might be ascribed to both pre- and post-implant complications, besides to *in vivo* mechanical stress/insults, as for non-coronary leaflets [43], [44], and/or transient hypoxic conditions during explant procedures, since suffering cells were also noticed in autografts. TRICOL treatment grants preservation of the adult basal lamina [18], [19]. Nonetheless, a differential detergent sensitivity of tissues with diverse post-natal developmental ages cannot be excluded and is yet an unaddressed appraisal for TEHV approaches based on pediatric tissues.

Reported outcomes on their biological performance revealed TRICOL heart valve scaffolds as suitable tissue-guided self-regenerating replacements. The ability to instruct cells to a correct differentiated function can be expected only from an integral ECM, providing specific matrikine signaling for their homing, integration and acquisition of specific phenotypes. TRICOL substitutes do not elicit graft-versus-host disease and can sustain cell repopulation and ECM remodeling for graft adaptation to cardiac hemodynamics and somatic development.

Results from the present study fully support the concept of *in vivo* spontaneous guided tissue regeneration, conditioning decellularized valve allografts to viable and functional autologous-like replacements. Long-term outcomes observed for TRICOL aortic grafts in RVOT reconstruction are also encouraging allogeneic human clinical trials based on a similar rationale, overcoming the current paucity of pediatric size-pulmonary allografts. Plus, as alpha-gal free replacements, they foster human-like xenogeneic animal models to launch a new surgical therapeutic era, based on more physiological alternatives especially for pediatric patients.

## Supporting Information

Figure S1 **Quantitative immunophenotypical profile of allografted and autografted heart valves at 15 months from implantation.** For each antigen, expression has been considered in allograft (A, dot pattern) and in autograft (B, stripe pattern). Calcifying cells (von Kossa and OC), inflammatory and immune cell markers (CD45, Mast cells, CD68 and IL-10), EC markers (vWf and CD31), other vascular differentiated cell epitopes (MyHC-Apla1, vimentin, OPN, SMA, Calponin, smoothelin and SM-MyHC), ECM synthetizing cells (Pro-collagen I), haematopoietic stem cells (CD34 and CD117), mesenchymal stem cells (CD29, CD90 and CD105), embryonic stem cells (SSEA4 and OCT4) and neural stem cells (NGFr, GFAP and Nestin) are identified respectively by the shades of bordeaux red, dark blue, olive green, purple, orange, light blue, red, light green and black. In a., note the graphical elaboration for the *intima*, in b. for the *media*, in c. for the *adventitia* and in d. for the leaflet.
(DOC)

Figure S2 **Proliferative processes on allograft walls.** Note the high positivity and widespread distribution of PH3 in *intima* (A), *media* (B) and especially *adventitia* (C). Magnification 100 µm.
(DOC)

Table S1 Quantitative analyses on explanted decellularized aortic allografts and autogeneic RVOTs after 15 months of follow-up.
(DOC)

Table S2 Up- and- down-regulated genes in biological processes and cell components of valve allografts after 15 months.
(DOC)

Checklist S1 **ARRIVE guidelines checklist.**
(DOC)
