# Correction: Decellularized Allogeneic Heart Valves Demonstrate Self-Regeneration Potential after a Long-Term Preclinical Evaluation

**DOI:** 10.1371/journal.pone.0107601

**Published:** 2014-09-08

**Authors:** The PLOS ONE Staff

The legend for Figure 1 is incorrect. The correct legend can be viewed here.

**Figure 1:**
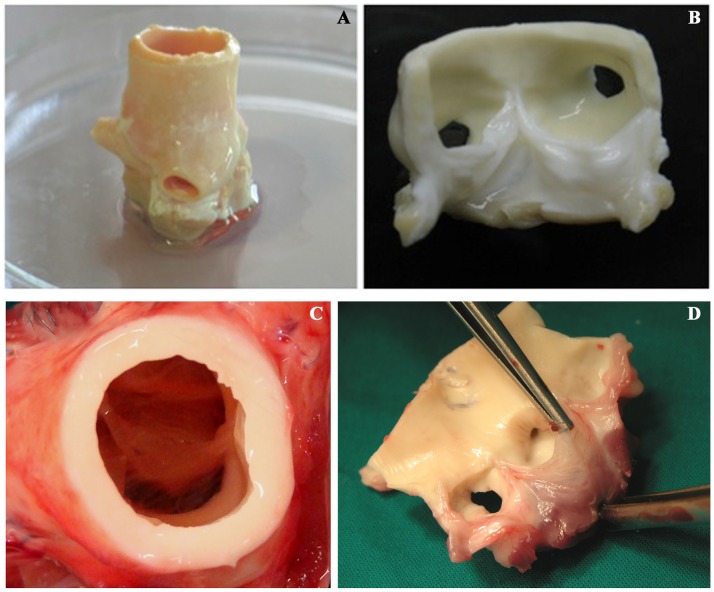
Macroscopic appearance of TRICOL allogeneic aortic valve before and after implantation. Allogeneic substitutes demonstrated similar gross morpho-anatomic structure to native valves without signs of leaflet fenestration, rupture or degeneration both after decellularization (A–B) and explant at 15 months (C–D).
