# Supplementary figures and images for: Decellularized Allogeneic Heart Valves Demonstrate Self-Regeneration Potential after a Long-Term Preclinical Evaluation
Source: PLoS One. 2014 Jun 18;9(6):e99593. doi: 10.1371/journal.pone.0099593 (PMC4062459; doi:10.1371/journal.pone.0099593)

**Supplementary Figure S1**

**a.**


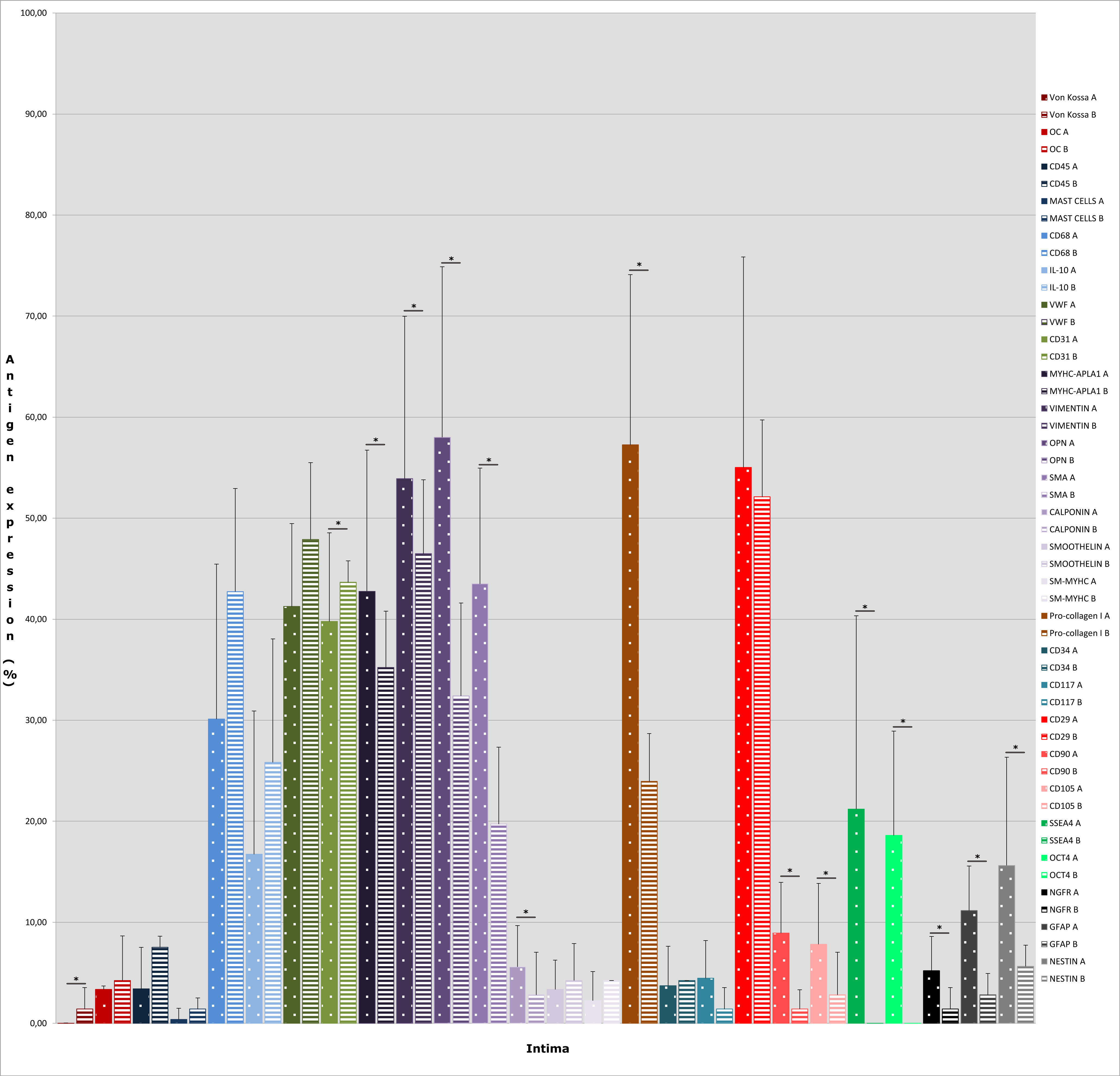


**b.**

**
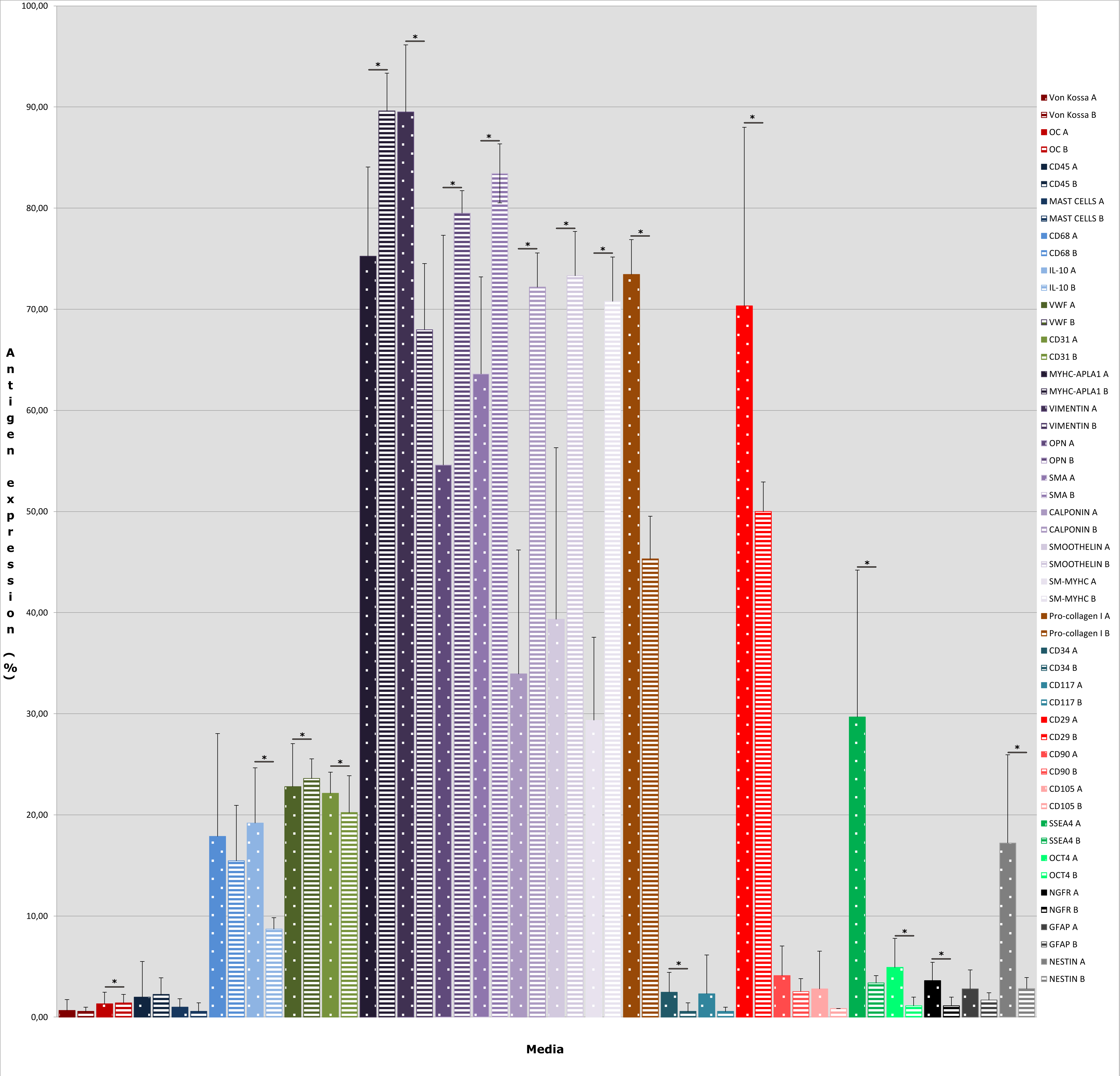
**

**c.**

**
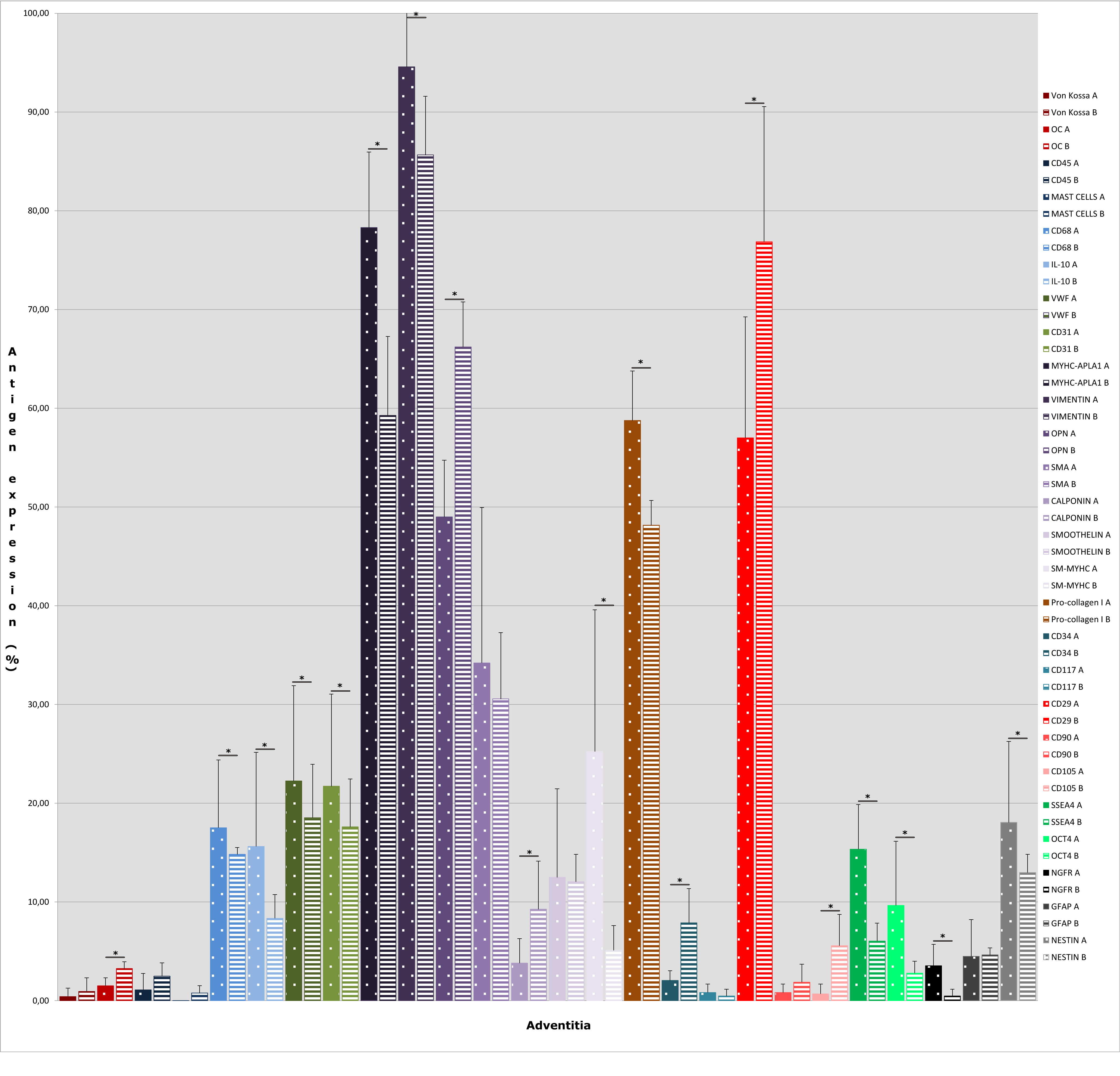
**

**d.**

**
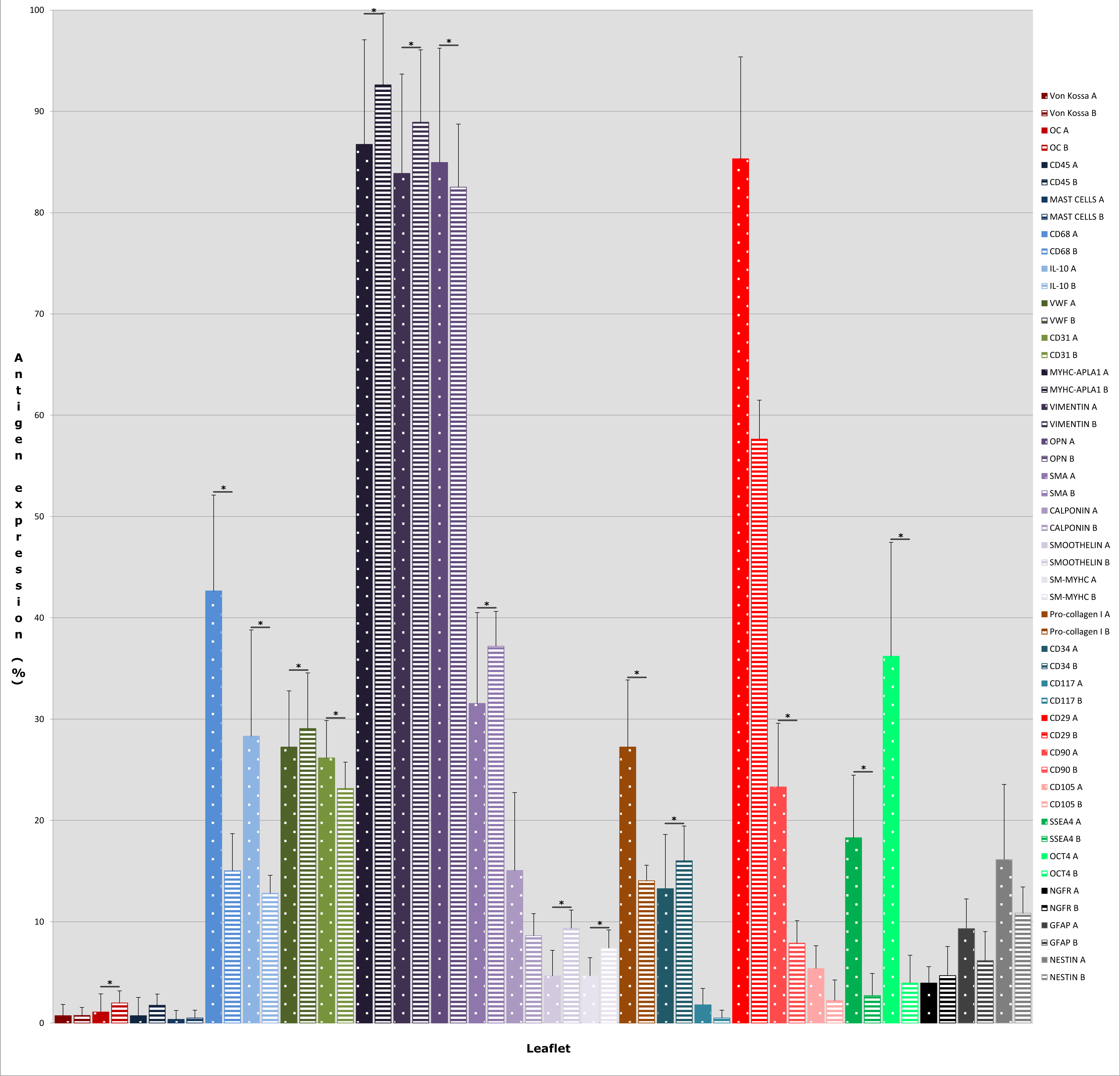
**

Supplement: Figure S1 — Quantitative immunophenotypical profile of allografted and autografted heart valves at 15 months from implantation. For each antigen, expression has been considered in allograft (A, dot pattern) and in autograft (B, stripe pattern). Calcifying cells (von Kossa and OC), inflammatory and immune cell markers (CD45, Mast cells, CD68 and IL-10), EC markers (vWf and CD31), other vascular differentiated cell epitopes (MyHC-Apla1, vimentin, OPN, SMA, Calponin, smoothelin and SM-MyHC), ECM synthetizing cells (Pro-collagen I), haematopoietic stem cells (CD34 and CD117), mesenchymal stem cells (CD29, CD90 and CD105), embryonic stem cells (SSEA4 and OCT4) and neural stem cells (NGFr, GFAP and Nestin) are identified respectively by the shades of bordeaux red, dark blue, olive green, purple, orange, light blue, red, light green and black. In a., note the graphical elaboration for the intima, in b. for the media, in c. for the adventitia and in d. for the leaflet. (DOC) [file pone.0099593.s001.doc]

**Supplementary Figure S2**


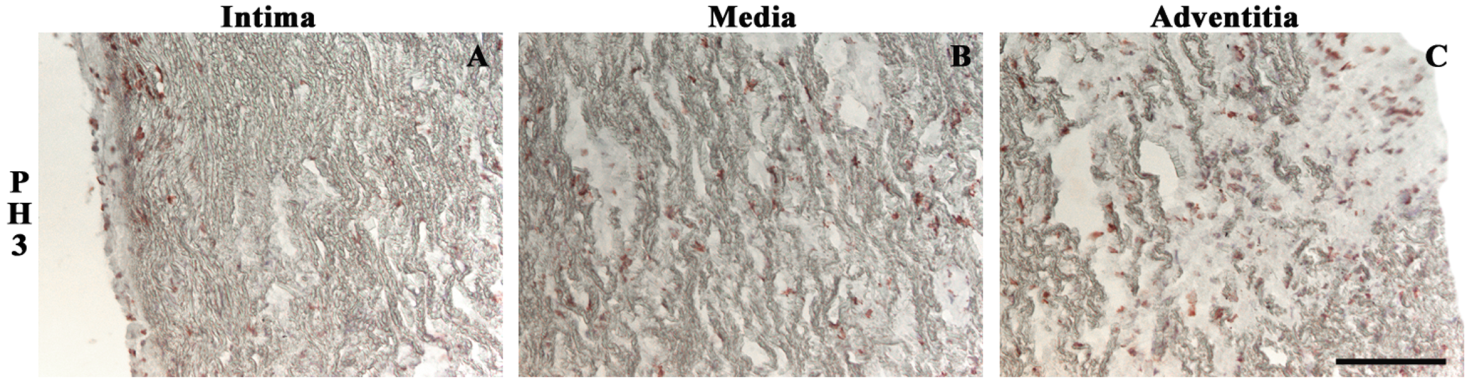

Supplement: Figure S2 — Proliferative processes on allograft walls. Note the high positivity and widespread distribution of PH3 in intima (A), media (B) and especially adventitia (C). Magnification 100 µm. (DOC) [file pone.0099593.s002.doc]
