# Supplementary material for: Decellularized Allogeneic Heart Valves Demonstrate Self-Regeneration Potential after a Long-Term Preclinical Evaluation
Source: PLoS One. 2014 Jun 18;9(6):e99593. doi: 10.1371/journal.pone.0099593 (PMC4062459; doi:10.1371/journal.pone.0099593)
Supplement: Table S1 — Quantitative analyses on explanted decellularized aortic allografts and autogeneic RVOTs after 15 months of follow-up. (DOC) [file pone.0099593.s003.doc]

**Supplementary Table S1. Quantitative analyses on explanted decellularized aortic allografts and autogeneic RVOTs after 15 months of follow-up**

|  |  | | **Tissue** | **Reconstructed RVOT**  **(Group A)** | |  | **Repositioned RVOT**  **(Group B)** | | **p** |
| --- | --- | --- | --- | --- | --- | --- | --- | --- | --- |
|  |  | |  | **MEAN** | **SD** |  | **MEAN** | **SD** |  |
| **Calcification aspects** | **von kossa** | I | | 0,00 | 0,00 |  | 1,41 | 2,11 | P<0.05 |
|  |  | M | | 0,66 | 1,07 |  | 0,56 | 0,42 | NS |
|  |  | A | | 0,41 | 0,86 |  | 0,93 | 1,39 | NS |
|  |  | L | | 0,72 | 1,11 |  | 0,74 | 0,81 | NS |
|  | **OC** | I | | 3,35 | 0,35 |  | 4,23 | 0,00 | NS |
|  |  | M | | 1,31 | 1,15 |  | 1,40 | 0,84 | P<0.05 |
|  |  | A | | 1,49 | 0,81 |  | 3,24 | 0,69 | P<0.05 |
|  |  | L | | 1,08 | 1,80 |  | 1,97 | 1,21 | P<0.05 |
| **Inflammation and Macrophage subpopulations** | **CD45** | I | | 3,41 | 4,43 |  | 7,51 | 4,11 | NS |
|  |  | M | | 1,97 | 3,54 |  | 2,25 | 0,42 | NS |
|  |  | A | | 1,09 | 1,67 |  | 2,47 | 1,35 | NS |
|  |  | L | | 0,72 | 1,80 |  | 1,75 | 1,11 | NS |
|  | **Mast cells** | I | | 0,37 | 1,12 |  | 1,41 | 2,11 | NS |
|  |  | M | | 0,98 | 1,65 |  | 0,56 | 0,84 | NS |
|  |  | A | | 0,00 | 0,00 |  | 0,77 | 0,73 | NS |
|  |  | L | | 0,36 | 0,88 |  | 0,49 | 0,76 | NS |
|  | **CD68** | I | | 30,11 | 15,33 |  | 42,72 | 10,23 | NS |
|  |  | M | | 17,87 | 10,18 |  | 15,45 | 5,48 | NS |
|  |  | A | | 17,50 | 6,88 |  | 14,81 | 0,69 | P<0.05 |
|  |  | L | | 42,65 | 9,46 |  | 15,02 | 3,67 | P<0.05 |
|  | **IL-10** | I | | 16,73 | 14,19 |  | 25,82 | 12,22 | NS |
|  |  | M | | 19,18 | 5,47 |  | 8,71 | 1,11 | P<0.05 |
|  |  | A | | 15,60 | 9,53 |  | 8,33 | 2,41 | P<0.05 |
|  |  | L | | 28,32 | 10,48 |  | 12,81 | 1,79 | P<0.05 |
| **Endothelial markers** | **VWF** | I | | 41,26 | 8,20 |  | 47,89 | 7,62 | NS |
|  |  | M | | 22,79 | 4,24 |  | 23,60 | 1,93 | P<0.05 |
|  |  | A | | 22,25 | 9,63 |  | 18,52 | 5,42 | P<0.05 |
|  |  | L | | 27,24 | 5,55 |  | 29,06 | 5,50 | P<0.05 |
|  | **CD31** | I | | 39,78 | 8,76 |  | 43,66 | 2,11 | P<0.05 |
|  |  | M | | 22,13 | 2,09 |  | 20,22 | 3,65 | P<0.05 |
|  |  | A | | 21,71 | 9,32 |  | 17,59 | 4,86 | P<0.05 |
|  |  | L | | 26,16 | 3,70 |  | 23,15 | 2,59 | P<0.05 |
| **Arterial and valve differentiated markers** | **MyHC-Apla1** | I | | 42,75 | 13,97 |  | 35,21 | 5,59 | P<0.05 |
|  |  | M | | 75,25 | 8,82 |  | 89,61 | 3,75 | P<0.05 |
|  |  | A | | 78,29 | 7,64 |  | 59,26 | 8,01 | P<0.05 |
|  |  | L | | 86,74 | 10,33 |  | 92,61 | 7,10 | P<0.05 |
|  | **Vimentin** | I | | 53,90 | 16,08 |  | 46,48 | 7,32 | P<0.05 |
|  |  | M | | 89,51 | 6,64 |  | 67,98 | 6,54 | P<0.05 |
|  |  | A | | 94,57 | 6,02 |  | 85,65 | 5,93 | P<0.05 |
|  |  | L | | 83,87 | 9,81 |  | 88,92 | 7,14 | P<0.05 |
|  | **OPN** | I | | 57,99 | 16,90 |  | 32,39 | 9,21 | P<0.05 |
|  |  | M | | 54,59 | 22,71 |  | 79,49 | 2,23 | P<0.05 |
|  |  | A | | 48,98 | 5,74 |  | 66,20 | 4,55 | P<0.05 |
|  |  | L | | 84,95 | 11,28 |  | 82,51 | 6,23 | P<0.05 |
|  | **SMA** | I | | 43,49 | 11,47 |  | 19,72 | 7,62 | P<0.05 |
|  |  | M | | 63,61 | 9,60 |  | 83,43 | 2,92 | P<0.05 |
|  |  | A | | 34,19 | 15,73 |  | 30,56 | 6,70 | NS |
|  |  | L | | 31,54 | 8,99 |  | 37,19 | 3,42 | P<0.05 |
|  | **Calponin** | I | | 5,58 | 4,10 |  | 2,82 | 4,23 | P<0.05 |
|  |  | M | | 33,93 | 12,26 |  | 72,19 | 3,37 | P<0.05 |
|  |  | A | | 3,80 | 2,48 |  | 9,26 | 4,86 | P<0.05 |
|  |  | L | | 15,05 | 7,69 |  | 8,62 | 2,18 | NS |
|  | **Smoothelin** | I | | 3,35 | 2,90 |  | 4,23 | 3,66 | NS |
|  |  | M | | 39,34 | 16,97 |  | 73,31 | 4,38 | P<0.05 |
|  |  | A | | 12,48 | 8,97 |  | 12,04 | 2,78 | NS |
|  |  | L | | 4,66 | 2,51 |  | 9,36 | 1,79 | P<0.05 |
|  | **SM-MyHC** | I | | 2,23 | 2,90 |  | 4,23 | 0,00 | NS |
|  |  | M | | 29,34 | 8,23 |  | 70,79 | 4,38 | P<0.05 |
|  |  | A | | 25,24 | 14,33 |  | 5,09 | 2,50 | P<0.05 |
|  |  | L | | 4,66 | 1,62 |  | 7,39 | 1,87 | P<0.05 |
| **ECM neosynthesis** | **Pro-collagen I** | I | | 57,25 | 16,85 |  | 23,94 | 4,72 | P<0.05 |
|  |  | M | | 73,44 | 3,34 |  | 45,32 | 4,21 | P<0.05 |
|  |  | A | | 58,75 | 5,01 |  | 48,15 | 2,50 | P<0.05 |
|  |  | L | | 27,24 | 6,62 |  | 14,04 | 1,55 | P<0.05 |
| **Stem cell markers** | **SSEA4** | I | | 21,19 | 19,15 |  | 0,00 | 0,00 | P<0.05 |
|  |  | M | | 29,77 | 14,52 |  | 3,37 | 0,73 | P<0.05 |
|  |  | A | | 15,33 | 4,53 |  | 6,02 | 1,84 | P<0.05 |
|  |  | L | | 18,28 | 6,20 |  | 2,71 | 2,18 | P<0.05 |
|  | **OCT4** | I | | 18,59 | 10,33 |  | 0,00 | 0,00 | P<0.05 |
|  |  | M | | 4,92 | 2,86 |  | 1,12 | 0,84 | P<0.05 |
|  |  | A | | 9,63 | 6,50 |  | 2,78 | 1,20 | P<0.05 |
|  |  | L | | 36,20 | 11,25 |  | 3,94 | 2,75 | P<0.05 |
|  | **CD34** | I | | 3,72 | 3,90 |  | 4,23 | 0,00 | NS |
|  |  | M | | 2,46 | 1,95 |  | 0,56 | 0,84 | P<0.05 |
|  |  | A | | 2,04 | 1,62 |  | 7,87 | 3,47 | P<0.05 |
|  |  | L | | 13,26 | 5,34 |  | 16,01 | 3,42 | P<0.05 |
|  | **CD117** | I | | 4,46 | 3,74 |  | 1,41 | 2,11 | NS |
|  |  | M | | 230 | 3,84 |  | 0,58 | 0,42 | NS |
|  |  | A | | 0,81 | 0,86 |  | 0,46 | 0,69 | NS |
|  |  | L | | 1,79 | 1,62 |  | 0,49 | 0,76 | NS |
|  | **CD29** | I | | 55,02 | 20,83 |  | 52,11 | 7,62 | NS |
|  |  | M | | 70,33 | 17,67 |  | 50,00 | 2,35 | P<0.05 |
|  |  | A | | 56,99 | 12,26 |  | 76,85 | 13,68 | P<0.05 |
|  |  | L | | 85,30 | 10,06 |  | 57,64 | 3,85 | NS |
|  | **CD90** | I | | 8,92 | 5,02 |  | 1,41 | 2,11 | P<0.05 |
|  |  | M | | 4,10 | 2,93 |  | 2,53 | 1,26 | NS |
|  |  | A | | 0,81 | 0,86 |  | 1,85 | 1,84 | NS |
|  |  | L | | 23,30 | 6,29 |  | 7,88 | 2,22 | P<0.05 |
|  | **CD105** | I | | 7,81 | 6,03 |  | 2,82 | 4,23 | P<0.05 |
|  |  | M | | 2,79 | 3,72 |  | 0,84 | 0,00 | NS |
|  |  | A | | 0,68 | 1,08 |  | 5,56 | 3,18 | P<0.05 |
|  |  | L | | 5,38 | 2,26 |  | 2,22 | 2,04 | NS |
|  | **NGFr** | I | | 5,20 | 3,39 |  | 1,41 | 2,11 | P<0.05 |
|  |  | M | | 3,61 | 1,82 |  | 1,12 | 0,84 | P<0.05 |
|  |  | A | | 3,53 | 2,15 |  | 0,46 | 0,69 | P<0.05 |
|  |  | L | | 3,94 | 1,62 |  | 4,68 | 2,87 | NS |
|  | **GFAP** | I | | 11,15 | 4,43 |  | 2,82 | 2,11 | P<0.05 |
|  |  | M | | 2,79 | 1,87 |  | 1,69 | 0,73 | NS |
|  |  | A | | 4,48 | 3,71 |  | 4,63 | 0,69 | NS |
|  |  | L | | 9,32 | 2,94 |  | 6,16 | 2,87 | NS |
|  | **Nestin** | I | | 15,61 | 10,73 |  | 5,63 | 2,11 | P<0.05 |
|  |  | M | | 17,22 | 8,37 |  | 2,80 | 1,11 | P<0.05 |
|  |  | A | | 18,05 | 8,19 |  | 12,96 | 1,84 | P<0.05 |
|  |  | L | | 16,13 | 7,42 |  | 10,84 | 2,59 | NS |

Mean and SD values are expressed in percentage after normalization to counted nuclei.

Tissue classification: I=*Intima*; M= *Media*; A=*Adventitia* and L=Leaflet.

Among inflammatory cell markers tested, the counting for NK, T cells and B cells was not reported, as no positivity was detected, compatibly with histopathologic evaluations.

Stem immunophenotyping of engrafted/resident cells comprises markers expressed by several lineages, i.e. haematopoietic (CD34 and CD117), mesenchymal (CD29, CD90 and CD105), embryonic (SSEA4 and OCT4) and nervous (NGFr, GFAP and Nestin) ones.
