# Supplementary material for: Decellularized Allogeneic Heart Valves Demonstrate Self-Regeneration Potential after a Long-Term Preclinical Evaluation
Source: PLoS One. 2014 Jun 18;9(6):e99593. doi: 10.1371/journal.pone.0099593 (PMC4062459; doi:10.1371/journal.pone.0099593)
Supplement: Table S2 — Up- and- down-regulated genes in biological processes and cell components of valve allografts after 15 months. (DOC) [file pone.0099593.s004.doc]

**Supplementary Table S2. *Up- and- down-regulated genes in biological processes and cell components of valve allografts after 15 months***

| ***Rank*** | ***Term*** | ***p*** | ***Gene IDs*** |
| --- | --- | --- | --- |
| **Biological Process (Down-regulated genes in A.L. 15, clusters 1 and 3)** | | | |
| 1 | Blood coagulation (GO:0007596) | 2,36E-02 | PROCR, COL3A1, SERPING1 |
| 2 | Positive regulation of cell proliferation (GO:0008284) | 2,24E-02 | CTGF, AQP1, NAP1L1 |
| 3 | Aging (GO:0007568) | 1,11E-03 | CTGF, DCN, FOS |
| 4 | Cell surface receptor linked signaling pathway (GO:0007166) | 2,64E-02 | COL3A1, CTGF, FOS |
| 5 | Response to stress (GO:0006950) | 2,05E-02 | CTGF, FOS, AQP1 |
| 6 | Response to steroid hormone stimulus (GO:0048545) | 7,84E-03 | CTGF, FOS, AQP1 |
| 7 | Response to organic substance (GO:0010033) | 1,72E-02 | CTGF, FOS, AQP1 |
| 8 | Protein modification process (GO:0006464) | 1,96E-02 | COL3A1, DCN, FKBP7 |
| 9 | Response to abiotic stimulus (GO:0009628) | 1,04E-03 | DCN, FOS, AQP1 |
| 10 | Response to external stimulus (GO:0009605) | 1,04E-03 | DCN, FOS, AQP1 |
| 11 | Response to cytokine stimulus (GO:0034097) | 1,50E-02 | COL3A1, FOS |
| 12 | Wound healing (GO:0042060) | 8,49E-03 | COL3A1, DCN |
| **Biological Process (Up-regulated genes in A.L. 15, cluster 4)** | | | |
| 1 | Cell proliferation (GO:0008283) | 2,65E-03 | CKS2, LIPA, PRDX1, BST2, TSPO, TXN |
| 2 | Multicellular organismal process (GO:0032501) | 1,26E-02 | CSTB, APOE, MEF2D, MSX2, SPP1 |
| 3 | Regulation of cell proliferation (GO:0042127) | 1,00E-02 | GPX1, APOE, ARG1, MMP12, TSPO |
| 4 | Cell-cell signaling (GO:0007267) | 1,70E-02 | GRB2, ITGB2, BST2, TXN |
| 5 | Regulation of cellular process (GO:0050794) | 1,56E-03 | GPX1, PRDX1, TSPO, TXN |
| 6 | Epithelial cell proliferation (GO:0050673) | 7,10E-03 | GPX1, APOE, ARG1, MMP12 |
| 7 | Cellular catabolic process (GO:0044248) | 2,58E-04 | GPI, GPX1, APOE, PRDX1 |
| 8 | Cellular component movement (GO:0006928) | 5,84E-03 | ACTR3, TXN, CAPZA2 |
| 9 | Response to protein stimulus (GO:0051789) | 8,83E-03 | ACTR3, ARG1, MSX2 |
| 10 | Actin cytoskeleton organization (GO:0030036) | 1,55E-02 | ACTR3, ARPC1A, CAPZA2 |
| 11 | Response to hydrogen peroxide (GO:0042542) | 3,78E-03 | GPX1, ARG1, PRDX1 |
| 12 | Aging (GO:0007568) | 1,71E-02 | GRB2, APOE, TSPO |
| **Cellular Component (Down-regulated genes in A.L. 15, clusters 1 and 3)** | | | |
| 1 | Extracellular region (GO:0005576) | 1,94E-02 | COL3A1, CTGF, DCN, IGFBP6, MGP, SERPING1 |
| 2 | Extracellular space (GO:0005615) | 2,61E-03 | COL3A1, CTGF, DCN, IGFBP6, SERPING1 |
| 3 | Extracellular matrix (GO:0031012) | 8,08E-04 | COL3A1, DCN, MGP |
| 4 | Proteinaceous extracellular matrix (GO:0005578) | 2,68E-03 | CTGF, DCN, MGP |
| 5 | Cis-Golgi network (GO:0005801) | 1,66E-02 | CTGF |
| 6 | Oligosaccharyltransferase complex (GO:0008250) | 1,39E-02 | KRTCAP2 |
| 7 | Basal plasma membrane (GO:0009925) | 4,38E-02 | AQP1 |
| 8 | Symbiont-containing vacuole (GO:0020003) | 2,79E-03 | AQP1 |
| 9 | Symbiont-containing vacuole membrane (GO:0020005) | 2,79E-03 | AQP1 |
| 10 | Chromatin assembly complex (GO:0005678) | 8,35E-03 | NAP1L1 |
| 11 | MHC protein complex (GO:0042611) | 1,39E-02 | PROCR |
| 12 | Fibrillar collagen (GO:0005583) | 1,66E-02 | COL3A1 |
| **Cellular Component (Up-regulated genes in A.L. 15, cluster 4)** | | | |
| 1 | Cytoplasm (GO:0005737) | 3,90E-02 | ACTR3, ARPC1A, CSTB, GPI, GPX1, GRB2, KIF24, APOE, ARG1, ARG1, LGALS3, MSX2, PRDX1, PRKCI, RPS20, SPP1, TXN, CAPZA2 |
| 2 | Extracellular region (GO:0005576) | 7,58E-03 | IFI30, HAPLN1, GPI, CRB2, APOE, LGALS3, MMP12, SPP1, TXN, CAPZA2 |
| 3 | Cytosol (GO:0005829) | 3,94E-02 | GPI, GPX1, GRB2, ARG1, OAZ1, PRKCI, RPS20, TXN, CAPZA2 |
| 4 | Actin cytoskeleton (GO:0015629) | 1,45E-02 | ACTR3, ARPC1A, CAPZA2 |
| 5 | Proteinaceous extracellular matrix (GO:0005578) | 2,40E-02 | HAPLN1, LGALS3, MMP12 |
| 6 | Anchored to membrane (GO:0031225) | 2,77E-02 | BST2 |
| 7 | Late endosome (GO:0005770) | 9,85E-03 | APOE, BST2 |
| 8 | Extrinsic to external side of plasma membrane (GO:0031232) | 1,21E-02 | APOE |
| 9 | Chylomicron (GO:0042627) | 4,76E-02 | APOE |
| 10 | Polarisome (GO:0000133) | 6,07E-03 | PRKCI |
| 11 | F-actin capping protein complex (GO:0008290) | 1,81E-02 | CAPZA2 |
| 12 | Cortical cytoskeleton (GO:0030863) | 4,17E-02 | CAPZA2 |
| **Biological Process (Up-regulated genes in A.W. 15, cluster 2)** | | | |
| 1 | Muscle contraction (GO:0006936) | 6,06E-04 | CNN1, ACTC1, TPM2 |
| 2 | Actin filament-based process (GO:0030029) | 2,19E-05 | DSTN, ACTC1, TPM2 |
| 3 | Muscle organ development (GO:0007517) | 8,31E-03 | TAGLN, ACTC1 |
| 4 | Cell activation (GO:0001775) | 2,43E-02 | RHOB, SPARC |
| 5 | Vesicle-mediated transport (GO:0016192) | 4,76E-02 | RHOB, SPARC |
| 6 | Actin cytoskeleton organization (GO:0030036) | 1,05E-02 | CNN1, ACTC1 |
| 7 | Actin polymerization or depolymerization (GO:0008154) | 2,79E-02 | DSTN |
| 8 | Purine ribonucleoside triphosphate catabolic process (GO:0009207) | 4,23E-02 | ACTC1 |
| 9 | Negative regulation of DNA metabolic process (GO:0051053) | 1,48E-02 | TERF2IP |
| 10 | Regulation of purine nucleotide catabolic process (GO:0033121) | 2,30E-02 | TPM2 |
| 11 | Muscle cell development (GO:0055001) | 1,65E-02 | ACTC1 |
| 12 | Protein complex disassembly (GO:0043241) | 3,11E-02 | DSTN |

Genome-wide analyses revealed variations in expression for important genes involved in biological processes and cellular components both in leaflet and arterial walls of valve allografts.

A.L. 15: Allograft Leaflet after 15 implantation months

A.W. 15: Allograft Wall after 15 implantation months
